# Profile of serum lipid metabolites of one-week-old goat kids depending on the type of rearing

**DOI:** 10.1186/s12917-020-02575-1

**Published:** 2020-09-21

**Authors:** Michał Czopowicz, Agata Moroz, Olga Szaluś-Jordanow, Marcin Mickiewicz, Lucjan Witkowski, Tomasz Nalbert, Iwona Markowska-Daniel, Ryszard Puchała, Emilia Bagnicka, Jarosław Kaba

**Affiliations:** 1 Division of Veterinary Epidemiology and Economics, Institute of Veterinary Medicine, Warsaw University of Life Sciences, Nowoursynowska 159c, 02-776 Warsaw, Poland; 2 Department of Small Animal Diseases with Clinic, Institute of Veterinary Medicine, Warsaw University of Life Sciences, Nowoursynowska 159c, 02-776 Warsaw, Poland; 3 Applied Physiology Unit, Military Institute of Hygiene and Epidemiology, Kozielska 4, 01-001 Warsaw, Poland; 4 Institute of Genetics and Animal Breeding, Polish Academy of Sciences, Postępu 36A, Jastrzębiec, 05-552 Magdalenka, Poland

**Keywords:** Metabolomics, Snatching, Weaning

## Abstract

**Background:**

Weaning of goat kids immediately after birth and feeding them on bovine or heat-treated caprine colostrum, referred to as snatching, is considered an effective control measure in some infectious diseases. The study was carried out in one-week-old goat kids to gain insight into the profile of lipid metabolites and to investigate the influence of snatching on kids’ metabolism. Fifty-two goat kids born to 23 female goats were included in the study – 22 kids were weaned immediately after birth and kept isolated from their mothers; 30 remaining kids were left with their mothers for next 3 weeks so that they could nurse on dams’ milk at will. Blood was collected at the age of 1 week and serum was obtained by centrifugation. The concentration of lipid metabolites was determined with mass spectrometry using a commercial MxP® Quant 500 kit (Biocrates Life Sciences AG, Innsbruck, Austria).

**Results:**

Concentration of 240 lipid metabolites belonging to 10 lipid classes was above the limit of detection of the assay. These lipid metabolites were quantified and included in the analysis. Concentration of 2 lipid classes (acyl-alkyl-phosphatidylcholines and ceramides) and 31 lipid metabolites (14 triacylglycerols, 5 acyl-alkyl-phosphatidylcholines, 2 diacylphosphatidylcholines, 1 lyso-phosphatidylcholine, 5 ceramides, 2 sphingomyelins, and 2 cholesterol esters) differed significantly between the two groups of kids.

**Conclusion:**

Snatching of kids results in reduction of serum concentration of lipid metabolites, however, the magnitude of this phenomenon does not seem to be sufficient to negatively affect kids’ health condition. This study is the first in which the broad set of lipid metabolites of young ruminants was quantified using the novel metabolomic assay MxP® Quant 500 kit.

## Background

A close contact between a doe and kids straight after birth is essential for bonding [1]. Does actively lick the kids, which quickly stand up and seek the udder. The sooner they consume colostrum the more likely they are to acquire protective level of passive immunity against pathogens present in their surroundings. On the other hand, the lactogenic route of transmission plays vital role in the dissemination of some most important goat diseases, to mention only caprine arthritis-encephalitis (CAE) [2] and contagious agalactia [3]. Given that consumption of even a single portion of colostrum or milk is sufficient to give rise to the infection, weaning of kids immediately after birth and feeding them on bovine or heat-treated caprine colostrum, referred to as snatching, is considered an effective control measure [4–6]. This method has been shown to allow considerable reduction of the within-herd prevalence of small ruminant lentivirus infection (SRLV) [7], although personal commitment of a farmer responsible for goat management seems to play crucial role in the success of this program. Nevertheless, some observations indicate that snatching of kids may hamper their growth, yet the growth retardation appears to be only temporal. In our recent study [8] the difference in body weight of kids was apparent already at the age of 1 week and it gradually disappeared by the end of the third month of life.

Over the last decade metabolomics has made a considerable progress in investigating various aspects of livestock production and diseases, which has led to identification of biomarkers of bovine transition diseases such as mastitis, metritis, laminitis, hypocalcemia, ketosis [9, 10], displaced abomasum [11] and hepatic lipidosis [12] as well as production traits in dairy [13] and beef cattle [14]. On the other hand, metabolomic studies in small ruminants lagged far behind, with only a few studies so far carried out and focused on limited set of metabolites in adult male goats [15] and female goats [16] and sheep [17]. This situation resulted from the lack of widely available methods of quantification of most important metabolites on one side, and from the lesser role played by sheep and goats in the industry of most of countries on the other. Recent progress in metabolomics allowed to overcome the former obstacle, providing a commercial method allowing for quantification of a very wide range of metabolites, including metabolites belonging to all main classes of lipids in a small volume of a biological fluid. This is particularly important as a considerable part or in some conditions even the vast majority of biologically significant biomarkers have proven to be lipid metabolites [12]. Some recent studies in small ruminants quantified a limited spectrum of lipid metabolites in adult goats [15, 16], however the physiological concentration of them remains unknown in young small ruminants [18].

Therefore, we decided to conduct the study to quantify the full profile of lipid metabolites of very young goat kids and by this occasion to gain insight into the influence of separation from mothers and artificial feeding on kids’ metabolism.

## Results

Distribution of males and females was not balanced between groups – of 30 kids left with mothers only 2 were females (7%) while there were 15 females among 22 kids weaned immediately after birth (68%) (*p* < 0.001) (Table S1). As a consequence, at the age of 1 week kids in the former group were significantly heavier (median 5.3 kg, IQR 5.0 to 6.1 kg) than kids from the latter group (median 4.6 kg, IQR 4.3 to 5.0 kg) (*p* < 0.001). The groups did not differ significantly in term of the litter size (*p* = 0.552) – most of them were twins (50 and 54%, respectively), followed by triplets (30 and 37%, respectively), and singletons (20 and 9%, respectively).

**Fig. 1:**
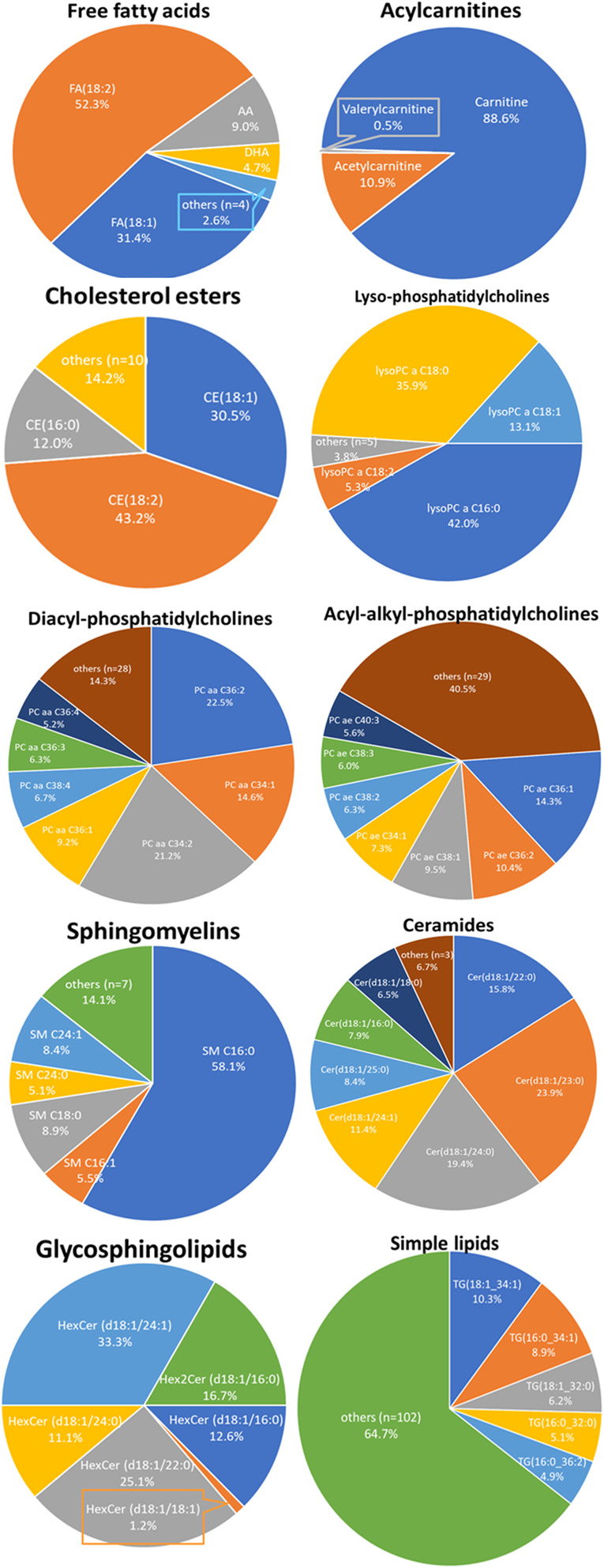
The share of main lipid metabolites in 10 lipid classes. Metabolites accounting for at least 5% of all metabolites in the class are shown, the rest are merged in the category of “others”

The concentrations of 240 lipid metabolites (44.9% of all 535 lipid metabolites covered by the assay) were above the limit of detection (LOD) of the assay and were included in the analysis (Tables S2 and S4): 8 free fatty acids (67% of 12), 3 acylcarnitines (8% of 40), 13 cholesteryl esters (59% of 22), 9 lyso-phosphatidylcholines (choline lyso-lecithins) (64% of 14), 35 diacyl-phosphatidylcholines (choline lecithins) (92% of 38), 36 acyl-alkyl-phosphatidylcholines (choline plasmalogens) (95% of 38), 12 sphingomyelins (80% of 15), 10 ceramides (36% of 28), 5 hexosylceramides (26% of 19), 1 dihexosylceramides (11% of 9), 0 trihexosylceramides (0% of 6), 1 diacylglicerol (diglyceride) (2% of 44), 106 triacylglycerols (triglycerides) (44% of 242), and choline. Several metabolites predominated in each lipid class (Fig. 1).

Phospholipids (mainly glycerophospholipids) and cholesterol esters were found at the highest concentrations in goat kids’ serum, ranging from 0.9 to 2.3 mmol/L and from 0.4 to 2.7 mmol/L, respectively, and each accounted for 35–40% of all serum lipids. Triacylglycerols and free fatty acids were next with concentrations varying from 0.2 to 2.2 mmol/L and from 0.3 to 1.4 mmol/L, respectively, and each constituted 10–15% of all lipids. Choline was present at the concentration ranging from 0.03 to 0.6 mmol/L and accounted for 2–6% of all lipids. Acylcarnitines constituted roughly 0.1% of all lipids. Of basic lipid classes two differed significantly between the two groups of kids – acyl-alkyl-phosphatidylcholines (*p* = 0.001) and ceramides (*p* = 0.002), and both had lower concentrations in kids weaned immediately after birth (Table 1).

**Table 1:** Main classes of lipids [μmol/L] in serum of one-week goat kids depending on the type of rearing

Class of lipids [μmol/L]	Kids weaned immediately after birth (*n* = 22)		Kids left with mothers (*n* = 30)		Mixed-effect ANCOVA	
	Median (IQR)	Range	Median (IQR)	Range	Type of rearing effect (*p*-value with Holm-Bonferroni correction)	Mother effect (*p*-value)
Free fatty acids^a^	419 (342–528)	271–714	566 (390–702)	272–1402	0.762	0.627
Acylcarnitines	16.4 (14.8–20.1)	10.3–26.7	24.3 (17.0–45.3)	12.2–92.2	0.222	0.020
Cholesterol esters^b^	1160 (1049–1492)	409–2703	1516 (1303–1910)	877–2256	0.396	0.062
Lysophosphatidylcholines (choline lyso-lecithins)	333 (246–385)	170–427	434 (290–515)	162–837	0.928	0.616
Diacyl-phosphatidylcholines (choline lecithins)	846 (708–1025)	551–1248	1003 (851–1112)	612–1351	0.064	0.053
Acyl-alkyl-phosphatidylcholines (choline plasmalogens)	122 (102–141)	71–177	154 (126–170)	82–236	0.001	0.001
Sphingomyelins	77 (68–83)	52–109	87 (80–99)	53–128	0.580	0.338
Ceramides	2.5 (1.8–2.9)	1.4–5.3	3.9 (2.9–5.1)	1.9–8.4	0.002	0.136
Glycosphingolipids	3.9 (3.3–4.4)	2.7–5.2	4.2 (3.8–4.8)	2.8–6.3	0.238	0.051
Simple lipids^c^	429 (303–612)	170–1518	662 (413–1081)	226–2214	0.566	0.261

^a^ to convert into mg/dL multiply by 0.0283

^b^ to convert into mg/dL multiply by 0.0386

^c^ to convert into mg/dL multiply by 0.0885

Altogether, 187 of 240 metabolites (77.9%) had positive fold change (FC_median_) which indicated that median concentration of a given metabolite in kids from the group left with mothers was higher than the median from the group weaned immediately after birth (Table S3). However, the criterion of FC_median_ above 1.5 was satisfied by 67 metabolites (27.9% of 240) and their concentrations were lower in the group weaned immediately after birth. Controlling for the effect of mother, size of the litter in which a kid was born and sex and body weight of a kid at the age of 1 week, 31 individual metabolites proved to differ significantly between kids weaned immediately after birth and kids left with mothers (12.9% of 240) (Table 2). These were: 14 triacylglycerols (45% of 31 metabolites), 8 phosphatidylcholines (26%) (5 acyl-alkyl-phosphatidylcholines, 2 diacylphosphatidylcholines and 1 lyso-phosphatidylcholine), 7 phosphosphingolipids (23%, of them 5 ceramides and 2 sphingomyelins), and 2 cholesterol esters (6%). The sex proved a significant confounder in two of those 31 metabolites – PC ae 38:4 (*p* = 0.033), and SM(OH) C16:1 (*p* = 0.036), and their concentrations were significantly higher in male kids. Neither litter size nor body weight turned out to be significantly linked to the concentration of any of those 31 metabolites. Out of lipid metabolites whose concentration was decreased in kids weaned immediately after birth only ceramides were significantly overrepresented (5 of 10 detected; 50%; *p* = 0.018), whereas the remaining constituted roughly 10–20% of all metabolites of this class detected: triacylglycerols (14 of 106; 13%), phosphatidylcholines (8 of 80; 10%), sphingomyelins (2 of 12; 17%) and cholesterol esters (2 of 13; 15%).

**Table 2:** Thirty one lipid metabolites whose concentration in serum [μmol/L] was significantly lower in one-week goat kids weaned immediately after birth

Metabolites	Kids weaned immediately after birth (*n* = 22)		Kids left with mothers (*n* = 30)		FC_median_^a^ (> 1.5)	Mixed-effect ANCOVA	
	Median (IQR)	Range	Median (IQR)	Range		Type of rearing effect (*p*-value with Holm-Bonferroni correction)	Mother effect (*p*-value)
**Cholesterol esters**							
CE(14:0)	15.2 (12.0–17.7)	7.41–29.7	24.8 (21.4–43.2)	15.8–68.0	1.64	0.015	0.035
CE(17:0)	8.08 (4.56–11.5)	1.75–32.6	28.3 (16.0–40.7)	6.17–58.1	3.50	0.015	0.003
**Lysophosphatidylcholines (choline lyso-lecithins)**							
lysoPC a C17:0	3.13 (2.06–4.34)	1.26–5.39	10.11 (7.41–12.3)	1.85–19.6	3.23	0.027	0.090
**Diacyl-phosphatidylcholines (choline lecithins)**							
PC aa C34:4	0.58 (0.45–0.72)	0.34–1.17	0.92 (0.75–1.23)	0.43–1.82	1.58	0.002	0.017
PC aa C36:1	55.7 (43.5–76.5)	33.6–105	109 (88.2–127)	33.3–194	1.95	0.001	0.007
**Acyl-alkyl-phosphatidylcholines (choline plasmalogens)**							
PC ae C34:0	1.02 (0.86–1.57)	0.63–1.89	5.18 (3.06–6.42)	0.77–8.65	5.07	< 0.001	0.003
PC ae C34:1	7.52 (6.62–8.27)	4.90–10.3	13.0 (9.60–14.3)	6.21–17.7	1.73	< 0.001	0.066
PC ae C36:0	0.80 (0.63–1.06)	0.48–1.55	2.18 (1.45–2.86)	0.66–3.88	2.73	< 0.001	0.009
PC ae C36:2	10.1 (7.60–13.0)	5.22–14.0	17.9 (13.6–21.5)	7.16–32.8	1.77	< 0.001	0.002
PC ae C38:4	3.54 (2.42–4.38)	1.78–5.29	6.80 (4.61–8.35)	2.28–12.6	1.92	0.030	< 0.001
**Sphingomyelins**							
SM (OH) C14:1	1.88 (1.57–2.09)	1.28–2.67	2.88 (2.36–3.64)	1.76–4.97	1.53	0.015	0.207
SM (OH) C16:1	1.25 (1.16–1.65)	0.81–2.28	3.03 (2.14–3.78)	1.20–5.53	2.43	0.015	0.002
**Ceramides**							
Cer(d18:1/16:0)	0.21 (0.16–0.23)	0.14–0.30	0.33 (0.24–0.40)	0.18–0.56	1.56	0.001	0.111
Cer(d18:1/18:0)	0.10 (0.08–0.15)	0.06–0.22	0.31 (0.19–0.40)	0.09–0.80	3.11	< 0.001	0.069
Cer(d18:1/22:0)	0.37 (0.26–0.48)	0.19–0.91	0.65 (0.40–0.90)	0.27–1.33	1.75	0.009	0.091
Cer(d18:1/23:0)	0.57 (0.37–0.68)	0.26–1.33	0.97 (0.61–1.30)	0.37–2.14	1.70	0.004	0.078
Cer(d18:1/25:0)	0.19 (0.15–0.23)	0.11–0.38	0.34 (0.27–0.44)	0.16–0.95	1.78	0.009	0.172
**Simple lipids**							
TG(14:0_36:1)	1.84 (1.33–2.35)	0.60–5.19	5.39 (3.66–8.53)	1.14–19.7	2.93	0.012	0.207
TG(16:0_28:1)	1.77 (1.28–2.20)	0.53–3.95	9.54 (5.64–19.8)	1.81–35.8	5.39	< 0.001	0.316
TG(16:0_28:2)	0.44 (0.37–0.53)	0.11–1.27	1.29 (0.81–2.79)	0.44–5.48	2.93	0.001	0.333
TG(16:0_33:1)	1.12 (0.65–2.05)	0.36–6.82	5.65 (3.33–8.35)	0.89–26.5	5.04	0.015	0.040
TG(16:0_35:1)	0.97 (0.54–1.35)	0.27–5.68	6.06 (3.76–8.40)	0.61–19.5	6.25	0.005	0.052
TG(17:0_34:1)	0.76 (0.53–1.22)	0.23–4.34	5.72 (3.66–7.85)	0.77–18.2	7.53	0.001	0.021
TG(17:0_34:2)	0.52 (0.41–0.81)	0.30–1.60	1.39 (0.89–1.95)	0.36–4.65	2.67	0.018	0.068
TG(18:0_32:1)	1.52 (0.93–2.19)	0.55–7.00	6.60 (3.82–11.6)	1.03–25.6	4.34	0.015	0.132
TG(18:1_26:0)	4.63 (3.39–6.36)	0.63–11.0	18.5 (9.57–31.7)	2.66–60.5	3.99	0.001	0.425
TG(18:1_28:1)	1.47 (1.01–1.99)	0.75–6.27	7.16 (4.65–13.4)	0.93–29.8	4.87	0.002	0.089
TG(18:1_31:0)	1.22 (0.73–2.28)	0.55–5.17	6.22 (4.11–8.74)	0.90–25.9	5.10	0.015	0.037
TG(18:1_33:0)	0.85 (0.51–1.43)	0.28–4.85	6.64 (4.33–9.27)	0.55–22.7	7.81	0.001	0.068
TG(18:1_33:1)	1.27 (0.61–1.69)	0.35–6.83	6.49 (3.51–8.31)	0.81–26.5	5.11	0.009	0.120
TG(18:2_35:1)	0.51 (0.32–1.06)	0.17–1.47	1.29 (0.73–1.64)	0.30–3.88	2.54	0.046	0.037

^a^ the fold change based on medians

## Discussion

Our study is the first so far carried out in animals to quantify a wide range of lipid metabolites belonging to main lipid classes. This was possible thanks to the fact that a novel metabolomic assay, MxP® Quant 500 Kit, has been developed recently by Biocrates Life Sciences AG. This assay covers over 500 lipid metabolites. This range of metabolites is much wider than covered by the AbsoluteIDQ® p180 Kit, which we used in our previous studies dedicated to the metabolomic profile of adult goats with SRLV infection [15, 16]. The MxP® Quant 500 Kit allows to investigate 8 additional lipid classes – ceramides, dihydroceramides, hexosylceramides, dihexosylceramides, trihexosylceramides, cholesteryl esters, diglycerides, and triacylglycerols. As a consequence, the current study provides complete and detailed view of the lipid profile of investigated animals. Most of data on serum lipid profile of ruminants available in the literature include only concentrations of the most basic lipid classes such as triacylglycerols and cholesterol and have been performed using different laboratory methods. Moreover, the phrase “serum lipid profile” usually refers to the concentration of cholesterol, triacylglycerols and several classes of lipoproteins. Therefore, direct comparisons with our results are difficult. In general, profile of serum lipid metabolites of one-week-old goat kids appears to be similar to the limited profile of adult goats which we investigated in our previous studies [15, 16] as well as to the profile of other ruminant species, except for the concentration of triacylglycerols which seems to be much higher than in adults goats [19], sheep and cattle [20, 21]. This is likely a direct consequence of intensive feeding with either milk or milk replacer, which both contain high concentrations of triacylglycerols [22].

We chose to analyze metabolomic profile of kids at the age of 1 week as this was the moment when some negative impact of immediate weaning on kids’ body weight had first been recorded [8]. In general, our study showed that many lipid metabolites seemed to be present at lower concentrations in kids weaned immediately after birth. However, strict criteria of evaluating statistical significance which we assumed in this study identified only two classes of lipid metabolites and only 13% of all metabolites as significantly less abundant in these kids’ serum. Such stringent approach was essential to control for a familywise error likely to occur when plenty of variables (metabolites) were compared between relatively few individuals. These two classes were choline plasmalogens and ceramides. In terms of individual lipid metabolites whose concentrations were reduced in kids weaned immediately after birth they represented major lipid classes at balanced level except for ceramides which were significantly overrepresented (concentration of 50% of ceramides was reduced). The meaning of serum choline plasmalogens and ceramide concentration for ruminants’ health is not clear. They are known to constitute important component of all biological membranes, especially in the nervous system and the heart [23]. They are also highly bioactive compounds involved in diverse cell processes, including between-cell interaction, cell proliferation, differentiation, and apoptosis element [24]. Some role of choline plasmalogens and ceramides in neurodegenerative diseases in humans has also been suspected [25, 26]. Anyway, reduction of the serum concentration of these metabolites is unlikely to be anyhow detrimental for goat kids, which is to some extent confirmed by a satisfactory performance of these kids in their further life as presented in our previous study [8]. In previous studies carried out in cattle most of biomarkers of metabolic disease were phosphatidylcholines and sphingomyelins, which for example constituted 69 and 24% of identified biomarkers of hepatic lipidosis, respectively [12]. The differences in metabolite concentration between the two groups of kids in our study overlapped with those observations only in terms of three choline plasmalogens (PC ae C34:1, PC ae C36:2, PC ae C38:4), and the majority of differing lipid metabolites were triacylglycerols and ceramides. This indicates that weaning of kids immediately after birth does not seem to have detrimental influence on their metabolism. However, the strong representation of triacylglycerols and ceramides may also result from the fact that our study was the first to investigate such a wide panel of lipid metabolites in livestock, as the novel assay provided by Biocrates company, which is the first to allow quantification of so many various triglicerides and ceramides, has never been used in animals before. Therefore, our study is likely to constitute an useful background for further analyses and comparisons when similarly broad spectra of lipid metabolites are quantified in other groups of livestock.

## Conclusions

Our study is the first to quantify and analyze the broad spectrum of lipid metabolites not only of goat kids but also of young ruminants in general. The immediate weaning of goat kids and rearing them on bovine colostrum and milk replacer results in reduction of serum concentration of some lipid metabolites, especially choline plasmalogens and ceramides, at the age of 1 week. Nevertheless, the magnitude of this phenomenon does not seem to be sufficient to negatively affect kids’ health condition.

## Methods

### Animals and blood collection

The study was carried out in the research goat herd of the Experimental Farm of the Institute of Genetics and Animal Breeding of Polish Academy of Sciences in 2014 and 2015. The herd had been infected with SRLV for over 20 years and was currently enforcing voluntary CAE control program based on immediate weaning of kids and raising them on bovine colostrum and milk replacer without contact with their dams [7].

In total 52 goat kids born to 23 does were included in the study – 32 kids born to 17 different does in 2014, and 20 kids born to 11 different does in 2015 which amounted to 28 parturitions. Five does gave birth to study kids in both subsequent years. Does aged between 3 and 9 years with the median (IQR) of 5 (4 to 6) years and belonged to the Polish White Improved (PWI, *n* = 17) or Polish Fawn Improved breed (PFI, *n* = 6).

In 28 parturitions 9 litters comprised singletons (32%), 14 twins (50%), and 5 triplets (18%). Of them 6, 7 and 4 litters, respectively, were delivered in 2014. Thirty five kids (67%) were males (20 born in 2014, 15 born in 2015), and 17 (33%) were females (12 born in 2014, 5 born in 2015). Of 52 kids born to study does 22 (42%) were weaned immediately after birth and kept isolated from their mothers. For the first 5 days of life kids were fed with bovine colostrum 150–250 ml 4 times a day through a nipple bottle. Subsequently, they were switched onto the milk replacer (Sprayfo Primo Goat Kid, Trouw Nutrition, Poland – composition according to the manufacturer: lactose 36.5%, protein 22%, fat 22%, vitamins & minerals 8%, moisture 3%), which was served from troughs 3 times a day at daily dose of 1 l. Thirty remaining kids (58%) were left with their mothers for next 3 weeks so that they could nurse on dams’ milk at will (Table S1).

Both kids and their mothers were clinically examined by board-certified specialists in small ruminant diseases (MC and JK, Diplomates of the European College of Small Ruminant Health Management) and all animals were apparently healthy. Kids were blood-sampled at the age of 1 week. Blood was taken from the jugular vein to 10 ml plastic tubes coated with clotting activator (BD Vacutainer, Becton Dickinson, Franklin Lakes, NJ, USA) and left overnight in the refrigerator to clot. Then, the serum was separated from the clot by centrifuging for 10 min at 3000 rpm, harvested into 2 ml aliquots, frozen and stored at − 80 °C for metabolomic analyses. Blood collection was carried out as a part of voluntary CAE surveillance and was approved by the 3rd Local Ethical Committee in Warsaw, Poland (Approval No. 31/2013, 22 May 2013).

### Metabolomic analysis

The concentration of metabolites was determined with mass spectrometry using a commercial MxP® Quant 500 kit (Biocrates Life Sciences AG, Innsbruck, Austria). The MxP® Quant 500 kit has been developed to quantify 630 endogenous metabolites belonging to 26 biochemical classes. Precisely, 523 metabolites are lipids belonging to 12 classes: acylcarnitines (40), lysophosphatidylcholines (14), phosphatidylcholines (76), sphingomyelins (15), ceramides (28), dihydroceramides (8), hexosylceramides (19), dihexosylceramides (9), trihexosylceramides (6), cholesteryl esters (22), diglycerides (44), triglycerides (242). Lipids were measured by flow injection analysis-tandem mass spectrometry (FIA-MS/MS) using a 5500 QTRAP® instrument (AB Sciex, Darmstadt, Germany) with an electrospray ionization source. The experimental metabolomics measurement technique is described in detail by patents EP1897014B1 and EP1875401B1 (accessible online at https://patents.google.com/patent/ EP1897014B1 and https://patents.google.com/patent/ EP1875401B1). Briefly, a 96-well based sample preparation device was used to quantitatively analyze the metabolite profile in the samples. This device consists of inserts that have been impregnated with internal standards, and a predefined sample amount was added to the inserts. Next, a phenyl isothiocyanate (PITC) solution was added to derive some of the analytes, and after the derivatization was completed, the target analytes were extracted with an organic solvent, followed by a dilution step. The obtained extracts were then analyzed by FIA-MS/MS method using multiple reaction monitoring to detect the analytes. Data were quantified using appropriate mass spectrometry software (Sciex Analyst®) and imported into Biocrates MetIDQ™ software for further analysis.

The analysis was performed in the laboratory of the Biocrates Life Sciences AG company in Innsbruck, Austria (Test report 5540 Sub2/2019) in June 2019. Concentrations of all metabolites were calculated in μM (μmol/L) and normalized with respect to the internal quality control samples. Only metabolites for which > 90% of measurements were above the limit of detection (LOD) were included in the further statistical analysis (missing measurements were substituted with the arithmetic mean of all valid measurements). This study focused on lipid metabolites. The list of all lipid metabolites included in the assay along with those statistically analyzed is given in Table S4.

### Classification of metabolites

Metabolites were classified into 3 basic groups: i) simple lipids built of glycerol esterified by fatty acids, ii) complex lipids composed of glycerol or another alcohol, fatty acids and an additional chemical compound, and iii) lipid precursors and derivatives (Fig. S5). However, for the needs of statistical analysis 10 lipid classes were distinguished to optimally represent compounds of different composition and function: free fatty acids (FFA), acylcarnitines (C), cholesterol esters (CE), lysophosphatidylcholines (choline lyso-lecithins, lyso-PC), diacyl-phosphatidylcholines (choline lecithins, PC aa), acyl-alkyl-phosphatidylcholines (choline plasmalogens, PC ae), sphingomyelins (SM), ceramides (Cer), glycosphingolipids (HexCer), and simple lipids.

The lipid side chain composition was abbreviated with “Cx:y”, where “x” signified the total carbon number of one or both side chains and “y” the total number of double bonds. In phosphatidylcholines letter “a” indicated the presence of ester bond while letter “e” indicated the presence of ether bond in the glycerol moiety. Two letters “aa” (=diacyl) and “ae” (=acyl-alkyl) indicated the presence of two fatty acid residues at the *sn-1* and *sn-2* position in the glycerol backbone, while a single letter “a” (=acyl) indicated the presence of a single fatty acid residue at the *sn-1* position in the glycerol backbone. On this basis PCs were classified as diacyl-phosphatidylcholines (PC aa x:y) (choline lecithins), acyl-alkyl-phosphatidylcholines (PC ae x:y) (choline plasmalogens), and lyso-phosphatidylcholines (lyso-PC a x:y) (choline lyso-lecithins). In cholesterol esters the number of double bonds and carbon atoms (y) present in the fatty acid residue (x) were denoted as “CE(x:y)”. In diacylglyceroles the number of carbon atoms (x and u) and double bonds (y and v) present in the fatty acid residues at *sn-1* and *sn-2* position, respectively, were denoted as “DG(x:y_u:v)”. In triacylglycerols the number of carbon atoms (x) and double bonds (y) present in the fatty acid residues at *sn-1* position and the total number of carbon atoms (n) and the total number of double bonds (m) of the two fatty acid residues at sn-2 and sn-3 position were denoted as TG(x:y_n:m). The “_” sign indicated that the positions (*sn-1*/*sn-2*/*sn-3*) of the fatty acid residues were unknown.

### Statistical analysis

Metabolite concentrations were presented as the median (Me), interquartile range (IQR) and range. The arithmetic mean and standard deviation (±SD) were also given for the overall concentration of metabolites (Table S2) to simplify the comparison with other studies and databases.

The differences in lipid classes and individual metabolite concentrations were evaluated between kids weaned immediately after birth (*n* = 22) and kids left with mothers for 3 weeks (*n* = 30) in two phases.

First, the fold change based on medians (FC_median_) was calculated according to the following formula, where Me_0_ and Me_1_ stood for the median in the immediately-after-birth weaned group and left with mothers group, respectively [27]:

$$ {\mathrm{FC}}_{\mathrm{median}}=\frac{{\mathrm{Me}}_1}{{\mathrm{Me}}_0}\ \mathrm{if}\ {\mathrm{Me}}_1>{\mathrm{Me}}_0\ \mathrm{or}\ {\mathrm{FC}}_{\mathrm{median}}=-\frac{{\mathrm{Me}}_0}{{\mathrm{Me}}_1}\mathrm{if}\ {\mathrm{Me}}_1<{\mathrm{Me}}_0 $$

Between-group difference in metabolite concentration was considered as possibly important if the absolute value of FC_median_ was above the absolute cut-off value of 1.5 [27].

Secondly, those metabolites which proved possibly important were compared between the two groups of kids using a mixed-effect analysis of covariance (ANCOVA). Before this step of analysis, normality of metabolite distribution was evaluated using the coefficient of skewness (CoS) with 95% confidence interval (CI 95%) and Shapiro-Wilk W test. CI 95% for the CoS was calculated as CoS ± 1.96×SE where SE was given by the formula:

$$ \mathrm{SE}=\sqrt{\frac{6\times \mathrm{n}\times \left(\mathrm{n}-1\right)}{\left(\mathrm{n}-2\right)\times \left(\mathrm{n}+1\right)\times \left(\mathrm{n}+3\right)}} $$

where n was sample size (i.e. 52 individual measurements). SE = 0.33. The distribution was considered as symmetric when CI 95% for the CoS covered 0. These metabolites which violated at least one criterion of normality (asymmetric distribution or significant result of the Shapiro-Wilks test) were transformed using the Box-Cox transformation [28] according to the formula:

$$ {\mathrm{y}}_{\mathrm{i}}^{\left(\uplambda \right)}=\left\{\begin{array}{c}\frac{{{\mathrm{y}}_{\mathrm{i}}}^{\uplambda}-1}{\uplambda}\ \mathrm{if}\ \uplambda \ne 0\\ {}\ln \left({\mathrm{y}}_{\mathrm{i}}\right)\kern1em \mathrm{if}\ \uplambda =0\end{array}\right. $$

The parameter λ was estimated using the profile likelihood function and varied from − 5 to 5.

The mixed-effect analysis of covariance (ANCOVA) initially included five independent variables: the variable “doe” (*D*) was fitted as a random effect and forced into the model to control for the lack of full independence of observations coming from related kids. The remaining 4 variables were fitted as fixed effects and retained in the model according to the backward stepwise elimination to control for potential confounding: the variable “sex” (S) to control for the imbalance between males and females between groups; the variable “litter size” (LS) to control for the influence of the number of kids born to one doe on their development; the covariate “body weight at 1 week” (BWT) to control for the imbalance in body weight between groups; and the main variable “immediately-after-birth weaning” (IW).

The concentration of a metabolite (y) estimated using ANCOVA was given by the following equation:

$$ y=\upmu +\mathrm{IW}+\left(\upbeta \times {\mathrm{X}}_{\mathrm{BWT}}+\mathrm{S}+\mathrm{LS}\right)+D+e $$

where μ signified the overall mean concentration of a metabolite, β – regression coefficient for the body weight, *D* – random effect of a mother (doe), and *e* – residual effect. IW, BWT, S, LW denoted the main fixed effects of variables. The variables in parentheses were potential confounders and covariates eliminated from the initial model according to the stepwise backward procedure. A familywise error was controlled by using Holm-Bonferroni correction of the *p*-value of the main variable (IW).

Categorical variables were presented as a count and percentage in groups and compared between groups using the Pearson’s chi-square test. Also, the distribution of metabolites whose concentration was affected by the type of rearing was evaluated with the Pearson’s chi-square test. A significance level (α) set at 0.05. All statistical tests were two-tailed. Statistical analysis was performed in TIBCO Statistica 13.3.0 (TIBCO Software Inc., Palo Alto, CA, USA).

## Supplementary information

**Additional file 1: Table S1.** Characteristics of 52 goat kids enrolled in the study. Description of the goat study population enrolled in this study.

**Additional file 2: Table S2.** Concentrations of 240 lipid metabolites in serum of one-week goat kids [μmol/L]. Results of the metabolomic analysis summarized for all 52 goat kids regardless of the type of rearing.

**Additional file 3: Table S3.** Concentrations of 240 lipid metabolites in serum of one-week goat kids depending on the type of rearing. Results of the metabolomic analysis summarized for all 52 goat kids with respect to the type of rearing.

**Additional file 4: Table S4.** Detailed results of metabolomic analysis of serum samples of 52 one-week-old goat kids using MxP® Quant 500 Kit. Detailed raw data analyzed in this study.

**Additional file 5: Figure S5.** Classification of lipid metabolites analyzed in this study. Graph showing the type of lipid metabolite classification used in this study.

## Data Availability

The data sets used and analyzed are available in Table S4.

## Abbreviations

ANCOVA: Analysis of covariance
BWT: Variable “body weight at the age of 1 week”
β: Regression coefficient
C: Acylcarnitines
CAE: Caprine arthritis-encephalitis
CE: Cholesterol esters
Cer: Ceramides
CI 95%: Confidence interval for the level of confidence of 95%
CoS: Coefficient of skewness
*D*: Random effect of the mother (doe)
DG: Diacylglycerol (diglyceride)
*e*: Residual effect
ESS: Error sum of squares
FFA: Free fatty acids
FC_median_: Fold change based on medians
HexCer: Hexosylceramides
Hex2Cer: Dihexosylceramides
Hex3Cer: Trihexosylceramides
IQR: Interquartile range
IW: Variable “weaning immediately after birth”
LOD: Limit of detection
LS: Variable “litter size”
lyso-PC: Lysophosphatidylcholines (choline lyso-lecithins)
Me: Median
μ: The overall mean concentration of a metabolite
PC aa: Diacyl-phosphatidylcholines (choline lecithins)
PC ae: Acyl-alkyl-phosphatidylcholines (choline plasmalogens)
PFI: Polish Fawn Improved breed
PWI: Polish White Improved breed
S: Variable “sex”
SD: Standard deviation
SE: Standard error
SM: Sphingomyelins
SRLV: Small ruminant lentivirus
TG: Triacylglycerols (triglycerides)
$$ \overline{\mathrm{x}} $$: Arithmetic mean
