# Supplementary material for: Profile of serum lipid metabolites of one-week-old goat kids depending on the type of rearing
Source: BMC Vet Res. 2020 Sep 21;16:346. doi: 10.1186/s12917-020-02575-1 (PMC7507259; doi:10.1186/s12917-020-02575-1)
Supplement: Supplementary file 1 — Additional file 1: Table S1. Characteristics of 52 goat kids enrolled in the study. Description of the goat study population enrolled in this study. [file 12917_2020_2575_MOESM1_ESM.docx]

Table S1. Characteristics of 52 goat kids enrolled in the study

| Kid | Sex | Mother | Serological status of the mother (year when positive for the first time) | Year of kid’s birth | Size of the litter in which the kid was born | Body weight at the age of 1 week [kg] | Weaning immediately after birth |
| --- | --- | --- | --- | --- | --- | --- | --- |
| 1 | male | 1 | positive (2007) | 2014 | singleton | 6.3 | yes |
| 2 | male | 2 | positive (2011) | 2014 | singleton | 5.0 | no |
| 3 | male | 3 | positive (2012) | 2014 | singleton | 4.6 | no |
| 4 | male |  |  | 2015 | singleton | 5.5 | no |
| 5 | male | 4 | positive (2014) | 2015 | twins | 6.1 | no |
| 6 | male |  |  | 2015 | twins | 7.7 | no |
| 7 | male | 5 | positive (2011) | 2014 | twins | 4.7 | no |
| 8 | male |  |  | 2014 | twins | 5.8 | no |
| 9 | female | 6 | positive (2011) | 2014 | twins | 5.1 | yes |
| 10 | female |  |  | 2014 | twins | 5.0 | yes |
| 11 | female | 7 | positive (2011) | 2014 | twins | 4.9 | yes |
| 12 | female |  |  | 2014 | twins | 5.0 | yes |
| 13 | female | 8 | negative | 2014 | triplets | 5.2 | yes |
| 14 | male |  |  | 2014 | triplets | 6.0 | yes |
| 15 | male |  |  | 2014 | triplets | 6.0 | yes |
| 16 | female | 9 | positive (2013) | 2014 | twins | 4.7 | yes |
| 17 | female |  |  | 2014 | twins | 3.8 | yes |
| 18 | male | 10 | negative | 2015 | twins | 3.7 | yes |
| 19 | male | 11 | positive (2011) | 2014 | twins | 5.1 | no |
| 20 | female |  |  | 2014 | twins | 4.6 | yes |
| 21 | male | 12 | positive (2013) | 2014 | twins | 5.1 | no |
| 22 | female |  |  | 2014 | twins | 3.9 | no |
| 23 | female |  |  | 2015 | twins | 4.5 | yes |
| 24 | female |  |  | 2015 | twins | 5.2 | yes |
| 25 | male | 13 | positive (2014) | 2015 | twins | 5.6 | no |
| 26 | male |  |  | 2015 | twins | 6.6 | no |
| 27 | male | 14 | negative | 2015 | twins | 4.9 | no |
| 28 | male |  |  | 2015 | twins | 5.0 | no |
| 29 | male | 15 | positive (2013) | 2014 | singleton | 3.9 | no |
| 30 | male |  |  | 2015 | triplets | 4.8 | no |
| 31 | male |  |  | 2015 | triplets | 4.6 | no |
| 32 | male | 16 | positive (2010) | 2014 | triplets | 6.1 | no |
| 33 | male |  |  | 2014 | triplets | 6.9 | no |
| 34 | male |  |  | 2014 | triplets | 7.0 | no |
| 35 | male | 17 | positive (2010) | 2014 | singleton | 7.3 | no |
| 36 | male | 18 | positive (2013) | 2014 | triplets | 5.4 | no |
| 37 | female |  |  | 2014 | triplets | 4.8 | yes |
| 38 | female |  |  | 2014 | triplets | 5.6 | yes |
| 39 | male | 19 | negative | 2015 | twins | 4.8 | yes |
| 40 | female |  |  | 2015 | twins | 5.0 | yes |
| 41 | male | 20 | positive (2013) | 2014 | twins | 4.5 | no |
| 42 | female |  |  | 2014 | twins | 4.8 | no |
| 43 | male |  |  | 2015 | twins | 4.6 | no |
| 44 | male |  |  | 2015 | twins | 5.3 | no |
| 45 | male | 21 | negative | 2014 | singleton | 4.3 | yes |
| 46 | male | 22 | positive (2013) | 2014 | triplets | 5.1 | yes |
| 47 | male |  |  | 2014 | triplets | 5.2 | no |
| 48 | male |  |  | 2014 | triplets | 5.2 | no |
| 49 | female |  |  | 2015 | triplets | 5.2 | yes |
| 50 | male |  |  | 2015 | triplets | 4.0 | no |
| 51 | female |  |  | 2015 | triplets | 4.7 | yes |
| 52 | male | 23 | positive (2013) | 2015 | singleton | 4.3 | no |
