# Supplementary material for: Profile of serum lipid metabolites of one-week-old goat kids depending on the type of rearing
Source: BMC Vet Res. 2020 Sep 21;16:346. doi: 10.1186/s12917-020-02575-1 (PMC7507259; doi:10.1186/s12917-020-02575-1)
Supplement: Supplementary file 2 — Additional file 2: Table S2. Concentrations of 240 lipid metabolites in serum of one-week goat kids [μmol/L]. Results of the metabolomic analysis summarized for all 52 goat kids regardless of the type of rearing. [file 12917_2020_2575_MOESM2_ESM.docx]

**Table S2.** Concentrations of 240 lipidic metabolites in serum of one-week goat kids [μmol/L]

| Metabolite | Mean ± SD | Median (IQR) | Range |
| --- | --- | --- | --- |
| Choline | 150.7 ± 88.49 | 127.9 (102.86 - 171.97) | 32.76 - 572.28 |
| **Free fatty acids** | | | |
| Arachidonic acid | 48.23 ± 33.92 | 36 (24.98 - 63.56) | 16.30 - 176.66 |
| Docosahexaenoid acid | 25.09 ± 11.65 | 23.15 (15.08 - 33.81) | 10.00 - 50.92 |
| Eicosapentaenoic acid | 2.80 ± 1.45 | 2.58 (1.80 - 3.56) | 1.07 - 9.33 |
| Octadecenoic acid | 168.6 ± 62.37 | 159.9 (117.9 - 187.4) | 82.64 - 360.21 |
| Octadecadienoic acid | 280.6 ± 141.1 | 242.7 (173.0 - 333.9) | 126.6 - 835.7 |
| Eicosenoic acid | 3.35 ± 1.36 | 3.18 (2.40 - 4.01) | 1.26 - 7.12 |
| Eicosadienoic acid | 2.73 ± 1.44 | 2.48 (1.74 - 3.10) | 1.20 - 9.09 |
| Eicosatrienoic acid | 5.02 ± 4.14 | 3.62 (2.42 - 6.63) | 1.51 - 25.88 |
| **Acylcarnitines** | | | |
| Carnitine | 23.31 ± 15.99 | 17.17 (13.92 - 24.3) | 9.42 - 79.66 |
| Acetylcarnitine | 2.87 ± 2.78 | 1.83 (1.24 - 3.12) | 0.74 - 14.78 |
| Valerylcarnitine | 0.13 ± 0.06 | 0.11 (0.08 - 0.14) | 0.06 - 0.37 |
| **Cholesterol esters** | | | |
| CE(14:0) | 25.00 ± 13.62 | 21.01 (15.72 - 30.82) | 7.41 - 68.04 |
| CE(15:0) | 9.31 ± 6.22 | 8.37 (4.12 - 12.37) | 2.33 - 26.42 |
| CE(16:0) | 174.0 ± 56.79 | 162.9 (142.3 - 195.8) | 49.35 - 303.44 |
| CE(17:0) | 20.82 ± 15.75 | 15.32 (8.03 - 30.58) | 1.75 - 58.10 |
| CE(18:0) | 16.45 ± 7.84 | 15.15 (10.2 - 20.54) | 2.80 - 36.07 |
| CE(18:1) | 441.1 ± 180.1 | 432.9 (309.2 - 534.9) | 62.39 - 981.4 |
| CE(18:2) | 624.5 ± 213.6 | 598.4 (471.4 - 722.9) | 228.9 - 1120.8 |
| CE(18:3) | 47.67 ± 20.11 | 45.40 (31.38 - 59.59) | 15.16 - 93.8 |
| CE(20:1) | 23.19 ± 16.26 | 18.78 (10.4 - 33.52) | 1.93 - 73.2 |
| CE(20:3) | 4.53 ± 1.55 | 4.44 (3.45 - 5.15) | 2.34 - 11.36 |
| CE(20:4) | 48.16 ± 26.62 | 43.29 (28.69 - 61.06) | 8.67 - 116.74 |
| CE(20:5) | 6.61 ± 2.48 | 6.17 (4.93 - 7.70) | 2.95 - 14.48 |
| CE(22:6) | 4.00 ± 2.30 | 3.42 (2.66 - 4.46) | 1.60 - 13.01 |
| **Lysophosphatidylcholines (choline lyso-lecithins)** | | | |
| lysoPC a C16:0 | 156.5 ± 58.01 | 152.9 (116.3 - 185.8) | 28.62 - 378.0 |
| lysoPC a C16:1 | 2.15 ± 0.99 | 2.02 (1.47 - 2.65) | 0.28 - 5.74 |
| lysoPC a C17:0 | 7.12 ± 5.19 | 5.14 (2.75 - 10.54) | 1.26 - 19.58 |
| lysoPC a C18:0 | 133.8 ± 57.79 | 131.1 (84.53 - 171.36) | 49.72 - 305.5 |
| lysoPC a C18:1 | 49.04 ± 21.61 | 47.15 (32.52 - 64.51) | 14.07 - 108.1 |
| lysoPC a C18:2 | 19.64 ± 8.96 | 19.41 (13.2 - 24.86) | 1.54 - 39.73 |
| lysoPC a C20:3 | 0.90 ± 0.32 | 0.85 (0.66 - 1.09) | 0.34 - 1.64 |
| lysoPC a C20:4 | 3.51 ± 2.02 | 3.06 (2.08 - 4.30) | 0.75 - 11.66 |
| lysoPC a C28:0 | 0.40 ± 0.18 | 0.34 (0.27 - 0.47) | 0.22 - 0.94 |
| **Diacyl-phosphatidylcholines (choline lecithins)** | | | |
| PC aa C24:0 | 0.29 ± 0.14 | 0.26 (0.19 - 0.36) | 0.12 - 0.73 |
| PC aa C28:1 | 1.27 ± 0.36 | 1.26 (0.98 - 1.40) | 0.80 - 2.44 |
| PC aa C30:0 | 5.44 ± 2.96 | 4.62 (3.68 - 6.68) | 1.91 - 18.74 |
| PC aa C32:0 | 25.43 ± 10.56 | 24.00 (17.72 - 29.59) | 14.25 - 73.24 |
| PC aa C32:1 | 9.39 ± 3.54 | 8.55 (7.38 - 11.02) | 4.03 - 20.58 |
| PC aa C32:2 | 3.79 ± 1.53 | 3.85 (2.78 - 4.94) | 1.10 - 7.19 |
| PC aa C32:3 | 0.41 ± 0.11 | 0.39 (0.33 - 0.51) | 0.24 - 0.67 |
| PC aa C34:1 | 138.8 ± 33.26 | 134.0 (121.0 - 162.8) | 67.58 - 220.2 |
| PC aa C34:2 | 201.4 ± 48.28 | 201.0 (167.1 - 223.0) | 125.9 - 343.0 |
| PC aa C34:3 | 7.82 ± 2.07 | 8.06 (5.95 - 9.32) | 3.82 - 12.41 |
| PC aa C34:4 | 0.84 ± 0.36 | 0.76 (0.54 - 1.13) | 0.34 - 1.82 |
| PC aa C36:0 | 4.27 ± 2.91 | 3.26 (2.12 - 5.78) | 1.12 - 14.9 |
| PC aa C36:1 | 87.39 ± 37.92 | 88.14 (54.32 - 110.8) | 33.29 - 194.32 |
| PC aa C36:2 | 213.5 ± 48.03 | 208.1 (182.2 - 247.8) | 118.2 - 317.06 |
| PC aa C36:3 | 59.37 ± 17.37 | 58.57 (43.81 - 71.84) | 27.58 - 92.79 |
| PC aa C36:4 | 48.86 ± 18.36 | 42.91 (33.89 - 60.99) | 23.44 - 93.86 |
| PC aa C36:5 | 4.25 ± 1.44 | 4.25 (3.05 - 4.98) | 1.98 - 9.00 |
| PC aa C36:6 | 0.27 ± 0.11 | 0.26 (0.21 - 0.35) | 0.07 - 0.58 |
| PC aa C38:0 | 4.06 ± 3.81 | 2.77 (1.62 - 4.76) | 0.73 - 20.84 |
| PC aa C38:1 | 1.65 ± 1.09 | 1.38 (0.87 - 2.00) | 0.42 - 5.63 |
| PC aa C38:3 | 13.33 ± 4.03 | 13.24 (9.51 - 16.12) | 6.38 - 23.43 |
| PC aa C38:4 | 63.18 ± 28.4 | 62.24 (36.56 - 74.92) | 25.00 - 131.2 |
| PC aa C38:5 | 22.07 ± 7.81 | 21.67 (14.43 - 28.9) | 10.27 - 39.2 |
| PC aa C38:6 | 6.30 ± 2.55 | 5.75 (4.45 - 7.99) | 2.34 - 12.35 |
| PC aa C40:2 | 2.34 ± 1.33 | 2.17 (1.34 - 3.25) | 0.27 - 5.90 |
| PC aa C40:3 | 2.01 ± 0.78 | 2.01 (1.42 - 2.42) | 0.75 - 4.10 |
| PC aa C40:4 | 4.26 ± 1.46 | 3.79 (3.18 - 5.06) | 1.88 - 7.47 |
| PC aa C40:5 | 8.38 ± 3.67 | 7.79 (5.53 - 10.9) | 3.03 - 19.98 |
| PC aa C40:6 | 5.11 ± 2.55 | 4.48 (3.02 - 6.86) | 1.55 - 12.22 |
| PC aa C42:0 | 0.44 ± 0.24 | 0.38 (0.27 - 0.59) | 0.10 - 1.27 |
| PC aa C42:1 | 0.34 ± 0.18 | 0.29 (0.21 - 0.44) | 0.09 - 0.87 |
| PC aa C42:2 | 0.42 ± 0.20 | 0.39 (0.27 - 0.53) | 0.10 - 0.84 |
| PC aa C42:4 | 0.52 ± 0.28 | 0.47 (0.30 - 0.69) | 0.12 - 1.27 |
| PC aa C42:5 | 0.41 ± 0.14 | 0.40 (0.30 - 0.48) | 0.14 - 0.72 |
| PC aa C42:6 | 0.46 ± 0.12 | 0.45 (0.36 - 0.53) | 0.25 - 0.81 |
| **Acyl-alkyl-phosphatidylcholines (choline plasmalogens)** | | | |
| PC ae C30:0 | 0.87 ± 0.36 | 0.74 (0.68 - 0.95) | 0.41 - 2.21 |
| PC ae C30:1 | 0.14 ± 0.05 | 0.14 (0.11 - 0.17) | 0.04 - 0.28 |
| PC ae C30:2 | 0.11 ± 0.04 | 0.10 (0.08 - 0.13) | 0.04 - 0.27 |
| PC ae C32:1 | 2.70 ± 0.68 | 2.64 (2.30 - 2.91) | 1.69 - 5.08 |
| PC ae C32:2 | 0.89 ± 0.19 | 0.84 (0.73 - 1.04) | 0.55 - 1.29 |
| PC ae C34:0 | 3.18 ± 2.38 | 1.85 (1.02 - 5.53) | 0.63 - 8.65 |
| PC ae C34:1 | 10.25 ± 3.59 | 9.19 (7.52 - 13.61) | 4.9 - 17.73 |
| PC ae C34:2 | 6.75 ± 1.81 | 6.26 (5.59 - 7.79) | 3.72 - 11.79 |
| PC ae C34:3 | 2.17 ± 0.62 | 2.05 (1.81 - 2.32) | 1.13 - 4.11 |
| PC ae C36:0 | 1.59 ± 0.92 | 1.27 (0.82 - 2.37) | 0.48 - 3.88 |
| PC ae C36:1 | 20.03 ± 6.56 | 19.28 (15.23 - 24.63) | 6.86 - 35.35 |
| PC ae C36:2 | 14.6 ± 6.19 | 13.50 (9.88 - 18.47) | 5.22 - 32.82 |
| PC ae C36:3 | 3.65 ± 1.01 | 3.61 (2.75 - 4.42) | 1.86 - 6.21 |
| PC ae C36:4 | 3.37 ± 1.38 | 2.93 (2.32 - 4.06) | 1.55 - 7.68 |
| PC ae C36:5 | 2.21 ± 1.04 | 1.96 (1.48 - 2.68) | 0.86 - 5.74 |
| PC ae C38:0 | 0.78 ± 0.40 | 0.69 (0.51 - 0.89) | 0.35 - 2.62 |
| PC ae C38:1 | 13.34 ± 6.37 | 11.91 (8.38 - 19.17) | 2.43 - 31.41 |
| PC ae C38:2 | 8.81 ± 3.83 | 7.68 (6.47 - 10.59) | 3.14 - 20.81 |
| PC ae C38:3 | 8.36 ± 3.23 | 7.68 (6.42 - 9.82) | 3.27 - 19.84 |
| PC ae C38:4 | 5.37 ± 2.80 | 4.73 (2.95 - 6.99) | 1.78 - 12.56 |
| PC ae C38:5 | 3.21 ± 1.38 | 2.91 (2.09 - 4.11) | 1.19 - 7.08 |
| PC ae C38:6 | 1.84 ± 0.87 | 1.72 (1.25 - 2.14) | 0.70 - 4.77 |
| PC ae C40:1 | 0.84 ± 0.35 | 0.80 (0.60 - 1.01) | 0.36 - 2.02 |
| PC ae C40:2 | 1.56 ± 0.58 | 1.46 (1.19 - 1.80) | 0.56 - 3.25 |
| PC ae C40:3 | 7.81 ± 4.16 | 7.37 (5.45 - 10.09) | 2.00 - 23.38 |
| PC ae C40:4 | 5.09 ± 2.04 | 4.55 (3.91 - 6.26) | 1.96 - 11.8 |
| PC ae C40:5 | 2.58 ± 1.01 | 2.39 (1.84 - 2.98) | 1.00 - 5.87 |
| PC ae C40:6 | 0.97 ± 0.54 | 0.83 (0.65 - 1.12) | 0.12 - 3.51 |
| PC ae C42:1 | 1.02 ± 0.59 | 0.90 (0.60 - 1.32) | 0.29 - 2.92 |
| PC ae C42:2 | 0.77 ± 0.42 | 0.72 (0.44 - 1.03) | 0.21 - 2.01 |
| PC ae C42:3 | 0.95 ± 0.39 | 0.92 (0.69 - 1.17) | 0.19 - 1.93 |
| PC ae C42:4 | 1.56 ± 0.87 | 1.54 (0.81 - 2.09) | 0.25 - 3.60 |
| PC ae C42:5 | 1.57 ± 0.59 | 1.45 (1.17 - 1.89) | 0.75 - 3.40 |
| PC ae C44:3 | 0.36 ± 0.16 | 0.32 (0.25 - 0.43) | 0.13 - 0.91 |
| PC ae C44:5 | 0.38 ± 0.13 | 0.37 (0.27 - 0.46) | 0.20 - 0.73 |
| PC ae C44:6 | 0.25 ± 0.08 | 0.23 (0.19 - 0.29) | 0.12 - 0.45 |
| **Sphingomyelins** | | | |
| SM (OH) C14:1 | 2.51 ± 0.90 | 2.35 (1.87 - 2.99) | 1.28 - 4.97 |
| SM (OH) C16:1 | 2.36 ± 1.23 | 2.04 (1.29 - 3.30) | 0.81 - 5.53 |
| SM (OH) C22:1 | 2.08 ± 0.47 | 2.02 (1.80 - 2.31) | 1.24 - 3.33 |
| SM (OH) C22:2 | 1.00 ± 0.29 | 1.02 (0.77 - 1.13) | 0.49 - 1.91 |
| SM (OH) C24:1 | 0.53 ± 0.13 | 0.54 (0.43 - 0.62) | 0.25 - 0.89 |
| SM C16:0 | 48.99 ± 9.71 | 48.59 (43.41 - 54.09) | 30.05 - 77.02 |
| SM C16:1 | 4.62 ± 1.09 | 4.45 (3.81 - 5.19) | 2.92 - 7.33 |
| SM C18:0 | 7.47 ± 2.00 | 7.19 (5.87 - 8.53) | 3.89 - 12.29 |
| SM C18:1 | 3.28 ± 0.92 | 3.07 (2.47 - 3.89) | 1.93 - 5.41 |
| SM C20:2 | 0.11 ± 0.04 | 0.10 (0.08 - 0.14) | 0.06 - 0.20 |
| SM C24:0 | 4.27 ± 0.95 | 4.25 (3.64 - 4.83) | 2.29 - 7.55 |
| SM C24:1 | 7.04 ± 2.10 | 6.69 (5.76 - 7.77) | 4.29 - 17.52 |
| **Ceramides** | | | |
| Cer(d16:1/23:0) | 0.09 ± 0.04 | 0.09 (0.07 - 0.11) | 0.03 - 0.20 |
| Cer(d16:1/24:0) | 0.08 ± 0.03 | 0.08 (0.06 - 0.10) | 0.03 - 0.15 |
| Cer(d18:1/16:0) | 0.28 ± 0.10 | 0.25 (0.21 - 0.35) | 0.14 - 0.56 |
| Cer(d18:1/18:0) | 0.23 ± 0.16 | 0.17 (0.10 - 0.33) | 0.06 - 0.80 |
| Cer(d18:1/20:0) | 0.07 ± 0.03 | 0.07 (0.05 - 0.08) | 0.03 - 0.14 |
| Cer(d18:1/22:0) | 0.56 ± 0.29 | 0.48 (0.33 - 0.71) | 0.19 - 1.33 |
| Cer(d18:1/23:0) | 0.84 ± 0.46 | 0.68 (0.50 - 1.16) | 0.26 - 2.14 |
| Cer(d18:1/24:0) | 0.68 ± 0.30 | 0.65 (0.47 - 0.86) | 0.26 - 1.71 |
| Cer(d18:1/24:1) | 0.40 ± 0.16 | 0.37 (0.29 - 0.49) | 0.16 - 0.98 |
| Cer(d18:1/25:0) | 0.29 ± 0.15 | 0.24 (0.19 - 0.39) | 0.11 - 0.95 |
| **Glycosphingolipids** | | | |
| HexCer (d18:1/16:0) | 0.52 ± 0.15 | 0.48 (0.42 - 0.60) | 0.26 - 0.96 |
| HexCer (d18:1/18:1) | 0.05 ± 0.01 | 0.05 (0.04 - 0.05) | 0.02 - 0.08 |
| HexCer (d18:1/22:0) | 1.04 ± 0.28 | 1.02 (0.82 - 1.26) | 0.56 - 1.76 |
| HexCer (d18:1/24:0) | 0.46 ± 0.12 | 0.44 (0.37 - 0.55) | 0.24 - 0.74 |
| HexCer (d18:1/24:1) | 1.37 ± 0.30 | 1.34 (1.16 - 1.51) | 0.84 - 2.32 |
| Hex2Cer (d18:1/16:0) | 0.69 ± 0.12 | 0.69 (0.61 - 0.75) | 0.42 - 1.00 |
| **Simple lipids** | | | |
| DG(16:0_18:2) | 1.16 ± 0.38 | 1.10 (0.90 - 1.37) | 0.40 - 2.18 |
| TG(14:0_32:2) | 1.43 ± 1.11 | 1.10 (0.76 - 1.51) | 0.27 - 5.32 |
| TG(14:0_34:0) | 5.15 ± 5.05 | 3.50 (1.89 - 6.15) | 0.68 - 22.57 |
| TG(14:0_34:1) | 18.59 ± 15.92 | 12.28 (9.24 - 23.64) | 2.15 - 76.82 |
| TG(14:0_34:2) | 4.83 ± 3.96 | 3.45 (2.35 - 5.47) | 0.69 - 18.65 |
| TG(14:0_36:1) | 4.65 ± 4.11 | 3.54 (1.80 - 6.62) | 0.60 - 19.66 |
| TG(14:0_36:2) | 8.04 ± 6.09 | 5.90 (3.73 - 11.17) | 0.85 - 32.54 |
| TG(14:0_36:3) | 2.68 ± 1.89 | 1.95 (1.41 - 3.10) | 0.32 - 9.32 |
| TG(16:0_28:1) | 8.28 ± 9.30 | 3.92 (1.87 - 10.89) | 0.53 - 35.78 |
| TG(16:0_28:2) | 1.27 ± 1.26 | 0.78 (0.45 - 1.51) | 0.11 - 5.48 |
| TG(16:0_30:2) | 3.47 ± 3.33 | 2.39 (1.23 - 4.49) | 0.60 - 17.32 |
| TG(16:0_32:0) | 35.05 ± 32.85 | 24.17 (15.72 - 41.08) | 4.76 - 177.3 |
| TG(16:0_32:1) | 24.53 ± 22.06 | 15.08 (11.79 - 32.33) | 2.97 - 113.27 |
| TG(16:0_32:2) | 4.95 ± 3.92 | 3.62 (2.42 - 5.78) | 0.68 - 18.93 |
| TG(16:0_32:3) | 0.59 ± 0.39 | 0.47 (0.35 - 0.60) | 0.16 - 1.77 |
| TG(16:0_33:1) | 4.85 ± 5.47 | 3.23 (1.04 - 5.99) | 0.36 - 26.52 |
| TG(16:0_33:2) | 1.16 ± 0.95 | 0.94 (0.59 - 1.24) | 0.25 - 4.75 |
| TG(16:0_34:0) | 14.79 ± 11.51 | 11.46 (7.75 - 16.84) | 3.85 - 56.67 |
| TG(16:0_34:1) | 61.93 ± 47.78 | 45.8 (30.59 - 69.38) | 9.12 - 243.21 |
| TG(16:0_34:2) | 20.6 ± 16.12 | 14.66 (9.99 - 24.64) | 3.69 - 70.18 |
| TG(16:0_34:3) | 2.53 ± 2.11 | 1.80 (1.13 - 2.87) | 0.51 - 8.51 |
| TG(16:0_35:1) | 4.59 ± 4.88 | 3.50 (0.97 - 6.47) | 0.27 - 19.47 |
| TG(16:0_35:2) | 2.74 ± 2.49 | 2.18 (0.89 - 3.80) | 0.22 - 10.61 |
| TG(16:0_35:3) | 0.66 ± 0.45 | 0.59 (0.35 - 0.77) | 0.15 - 2.15 |
| TG(16:0_36:2) | 33.74 ± 22.55 | 24.84 (18.91 - 45.09) | 5.73 - 94.31 |
| TG(16:0_36:3) | 14.82 ± 10.7 | 10.93 (6.77 - 17.68) | 3.01 - 43.56 |
| TG(16:0_36:4) | 3.15 ± 2.16 | 2.35 (1.55 - 3.97) | 0.82 - 8.74 |
| TG(16:0_38:1) | 0.56 ± 0.27 | 0.49 (0.41 - 0.63) | 0.19 - 1.62 |
| TG(16:0_38:2) | 1.17 ± 0.92 | 0.80 (0.58 - 1.48) | 0.28 - 4.79 |
| TG(16:0_38:3) | 1.00 ± 0.80 | 0.74 (0.55 - 1.15) | 0.28 - 4.86 |
| TG(16:0_38:4) | 1.26 ± 0.95 | 0.95 (0.61 - 1.50) | 0.42 - 4.97 |
| TG(16:0_38:5) | 1.03 ± 0.71 | 0.83 (0.54 - 1.18) | 0.19 - 3.35 |
| TG(16:1_28:0) | 1.70 ± 1.33 | 1.11 (0.90 - 2.13) | 0.43 - 6.27 |
| TG(16:1_30:1) | 1.01 ± 0.62 | 0.86 (0.61 - 1.15) | 0.34 - 3.24 |
| TG(16:1_32:0) | 4.01 ± 4.08 | 2.41 (1.77 - 4.32) | 0.46 - 22.27 |
| TG(16:1_32:1) | 2.68 ± 2.48 | 1.75 (0.99 - 3.49) | 0.4 - 11.69 |
| TG(16:1_34:0) | 1.81 ± 1.62 | 1.21 (0.66 - 2.44) | 0.24 - 7.11 |
| TG(16:1_34:1) | 7.20 ± 6.54 | 4.37 (3.18 - 8.61) | 0.87 - 31.38 |
| TG(16:1_34:2) | 2.26 ± 1.94 | 1.63 (1.05 - 2.30) | 0.47 - 8.60 |
| TG(16:1_36:1) | 1.36 ± 1.09 | 1.01 (0.62 - 1.98) | 0.20 - 5.18 |
| TG(16:1_36:2) | 3.08 ± 2.43 | 2.15 (1.45 - 3.97) | 0.65 - 11.33 |
| TG(16:1_36:3) | 1.20 ± 0.87 | 0.93 (0.66 - 1.42) | 0.34 - 4.45 |
| TG(17:0_34:1) | 4.24 ± 4.43 | 3.45 (0.79 - 5.98) | 0.23 - 18.22 |
| TG(17:0_34:2) | 1.26 ± 1.05 | 1.12 (0.52 - 1.59) | 0.30 - 4.65 |
| TG(17:1_34:1) | 2.05 ± 1.83 | 1.81 (0.64 - 2.61) | 0.23 - 8.89 |
| TG(18:0_30:0) | 5.92 ± 5.78 | 4.44 (1.92 - 7.02) | 1.00 - 25.69 |
| TG(18:0_30:1) | 2.84 ± 1.98 | 2.19 (1.61 - 3.64) | 0.30 - 9.09 |
| TG(18:0_32:1) | 5.67 ± 5.69 | 3.88 (1.52 - 7.59) | 0.55 - 25.61 |
| TG(18:0_32:2) | 1.24 ± 0.90 | 0.92 (0.70 - 1.58) | 0.29 - 4.32 |
| TG(18:0_34:2) | 3.65 ± 2.72 | 2.56 (1.89 - 4.80) | 0.63 - 11.44 |
| TG(18:0_34:3) | 0.71 ± 0.47 | 0.62 (0.36 - 0.75) | 0.11 - 2.28 |
| TG(18:0_36:3) | 3.46 ± 2.32 | 2.93 (1.96 - 4.43) | 0.72 - 10.24 |
| TG(18:0_36:4) | 0.94 ± 0.55 | 0.74 (0.54 - 1.12) | 0.27 - 2.52 |
| TG(18:1_26:0) | 14.15 ± 13.33 | 8.42 (5.24 - 19.99) | 0.63 - 60.48 |
| TG(18:1_28:1) | 6.27 ± 6.80 | 3.75 (1.47 - 8.65) | 0.75 - 29.76 |
| TG(18:1_30:0) | 24.06 ± 19.59 | 15.82 (12.07 - 30.3) | 2.39 - 87.22 |
| TG(18:1_30:1) | 12.52 ± 9.35 | 10.89 (5.89 - 16.33) | 1.26 - 48.5 |
| TG(18:1_30:2) | 2.08 ± 1.77 | 1.63 (0.93 - 2.48) | 0.28 - 9.20 |
| TG(18:1_31:0) | 5.17 ± 5.53 | 4.06 (1.16 - 6.57) | 0.55 - 25.94 |
| TG(18:1_32:0) | 42.88 ± 31.57 | 30.75 (21.74 - 49.31) | 6.47 - 151.63 |
| TG(18:1_32:1) | 23.54 ± 18.4 | 15.49 (11.38 - 33.64) | 3.23 - 93.55 |
| TG(18:1_32:2) | 3.98 ± 2.71 | 3.00 (2.00 - 5.08) | 0.62 - 14.01 |
| TG(18:1_33:0) | 4.85 ± 5.15 | 3.61 (0.85 - 7.06) | 0.28 - 22.67 |
| TG(18:1_33:1) | 4.86 ± 5.06 | 3.64 (1.20 - 7.01) | 0.35 - 26.53 |
| TG(18:1_33:2) | 0.98 ± 0.74 | 0.91 (0.50 - 1.08) | 0.22 - 4.34 |
| TG(18:1_34:1) | 71.59 ± 48.02 | 52.15 (38.35 - 102.97) | 11.18 - 218.64 |
| TG(18:1_34:2) | 19.75 ± 13.89 | 15.17 (9.62 - 22.45) | 3.81 - 58.09 |
| TG(18:1_34:3) | 2.02 ± 1.54 | 1.59 (1.02 - 2.28) | 0.41 - 7.43 |
| TG(18:1_35:2) | 2.46 ± 2.03 | 2.11 (0.89 - 3.46) | 0.23 - 11.24 |
| TG(18:1_36:3) | 10.28 ± 6.22 | 8.74 (5.94 - 10.87) | 3.65 - 30.43 |
| TG(18:1_36:4) | 1.99 ± 1.14 | 1.69 (1.12 - 2.45) | 0.76 - 5.96 |
| TG(18:1_36:5) | 0.38 ± 0.15 | 0.38 (0.27 - 0.42) | 0.14 - 0.87 |
| TG(18:1_38:5) | 0.90 ± 0.47 | 0.76 (0.52 - 1.22) | 0.32 - 2.15 |
| TG(18:2_28:0) | 4.96 ± 4.39 | 4.09 (2.00 - 6.25) | 0.45 - 22.29 |
| TG(18:2_30:0) | 5.24 ± 4.06 | 3.89 (2.50 - 6.14) | 0.78 - 18.44 |
| TG(18:2_30:1) | 2.25 ± 1.84 | 1.83 (1.02 - 3.04) | 0.26 - 9.47 |
| TG(18:2_31:0) | 1.43 ± 1.18 | 1.22 (0.63 - 1.48) | 0.35 - 5.23 |
| TG(18:2_32:0) | 10.13 ± 7.75 | 7.44 (4.79 - 12.24) | 1.99 - 29.85 |
| TG(18:2_32:1) | 4.42 ± 3.24 | 3.05 (2.26 - 5.54) | 0.71 - 15.03 |
| TG(18:2_32:2) | 0.88 ± 0.52 | 0.72 (0.52 - 0.98) | 0.23 - 2.40 |
| TG(18:2_33:1) | 0.99 ± 0.76 | 0.86 (0.43 - 1.21) | 0.17 - 4.08 |
| TG(18:2_34:0) | 4.32 ± 3.01 | 3.26 (2.3 - 4.99) | 0.93 - 12.06 |
| TG(18:2_34:1) | 16.49 ± 11.51 | 12.63 (8.04 - 20.37) | 3.48 - 47.88 |
| TG(18:2_34:2) | 4.65 ± 3.30 | 3.36 (2.43 - 6.1) | 1.06 - 14.53 |
| TG(18:2_35:1) | 1.06 ± 0.75 | 1.06 (0.41 - 1.41) | 0.17 - 3.88 |
| TG(18:2_36:0) | 0.74 ± 0.38 | 0.69 (0.47 - 0.85) | 0.17 - 2.06 |
| TG(18:2_36:1) | 3.54 ± 2.35 | 2.83 (1.92 - 4.81) | 0.73 - 10.52 |
| TG(18:2_36:2) | 5.53 ± 3.53 | 4.48 (2.99 - 6.27) | 1.81 - 17.47 |
| TG(18:3_34:1) | 1.34 ± 0.89 | 1.10 (0.74 - 1.53) | 0.30 - 3.93 |
| TG(18:3_36:2) | 0.55 ± 0.30 | 0.50 (0.36 - 0.63) | 0.19 - 1.68 |
| TG(20:1_34:1) | 1.17 ± 0.85 | 0.86 (0.59 - 1.50) | 0.30 - 4.45 |
| TG(20:2_34:1) | 0.69 ± 0.58 | 0.55 (0.35 - 0.75) | 0.11 - 3.54 |
| TG(20:3_32:0) | 1.20 ± 1.12 | 0.85 (0.59 - 1.34) | 0.18 - 7.42 |
| TG(20:3_32:1) | 0.73 ± 0.61 | 0.62 (0.40 - 0.76) | 0.11 - 3.42 |
| TG(20:3_34:1) | 2.30 ± 1.87 | 1.87 (1.11 - 2.82) | 0.29 - 10.43 |
| TG(20:3_34:2) | 0.77 ± 0.56 | 0.63 (0.41 - 0.96) | 0.15 - 3.42 |
| TG(20:3_36:3) | 0.57 ± 0.35 | 0.49 (0.37 - 0.65) | 0.22 - 2.59 |
| TG(20:4_30:0) | 0.70 ± 0.59 | 0.54 (0.39 - 0.72) | 0.20 - 3.02 |
| TG(20:4_32:0) | 1.43 ± 1.14 | 1.07 (0.72 - 1.55) | 0.29 - 5.10 |
| TG(20:4_32:1) | 0.68 ± 0.44 | 0.62 (0.33 - 0.79) | 0.19 - 1.93 |
| TG(20:4_34:0) | 0.84 ± 0.58 | 0.68 (0.50 - 0.90) | 0.17 - 2.52 |
| TG(20:4_34:1) | 2.15 ± 1.50 | 1.68 (1.00 - 2.91) | 0.46 - 6.07 |
| TG(20:4_34:2) | 0.88 ± 0.52 | 0.76 (0.47 - 1.07) | 0.20 - 2.39 |
| TG(20:4_36:2) | 1.07 ± 0.65 | 0.84 (0.59 - 1.53) | 0.30 - 3.08 |
| TG(20:4_36:3) | 0.60 ± 0.30 | 0.56 (0.38 - 0.74) | 0.19 - 1.49 |
| TG(22:5_32:0) | 0.63 ± 0.44 | 0.48 (0.36 - 0.67) | 0.15 - 2.59 |
| TG(22:5_34:1) | 0.69 ± 0.39 | 0.61 (0.40 - 0.77) | 0.20 - 1.85 |
