# Supplementary material for: Profile of serum lipid metabolites of one-week-old goat kids depending on the type of rearing
Source: BMC Vet Res. 2020 Sep 21;16:346. doi: 10.1186/s12917-020-02575-1 (PMC7507259; doi:10.1186/s12917-020-02575-1)
Supplement: Supplementary file 3 — Additional file 3: Table S3. Concentrations of 240 lipid metabolites in serum of one-week goat kids depending on the type of rearing. Results of the metabolomic analysis summarized for all 52 goat kids with respect to the type of rearing. [file 12917_2020_2575_MOESM3_ESM.docx]

**Table S3.** Concentrations of 240 lipidic metabolites in serum of one-week goat kids depending on the type of rearing

| Metabolite | Kids left with mothers (n=30) | | Kids weaned immediately after birth (n=22) | | FC_median_^a^ |  |
| --- | --- | --- | --- | --- | --- | --- |
|  | Median (IQR) | Range | Median (IQR) | Range |  |  |
| Choline | 145.3 (115.3-216.3) | 80.91-572.3 | 107.41 (93.53-141.1) | 32.76-176.3 | 1.35 |  |
| **Free fatty acids** | | | | | | |
| AA | 45.24 (27.45-75.6) | 18.13-176.7 | 32.37 (21.19-46.47) | 16.3-66.63 | 1.40 |  |
| DHA | 25.09 (16.93-34.09) | 10.46-50.92 | 17.37 (13.26-31.45) | 10.00-44.47 | 1.44 |  |
| EPA | 2.76 (1.95-3.83) | 1.11-9.33 | 2.50 (1.22-2.97) | 1.07-5.17 | 1.10 |  |
| FA(18:1) | 183.0 (144.6-235.1) | 84.09-360.2 | 138.4 (113.0-159.4) | 82.64-246.0 | 1.32 |  |
| FA(18:2) | 287.5 (194.7-378.6) | 131.8-835.7 | 216.1 (168.5-299.7) | 126.6-434.5 | 1.33 |  |
| FA(20:1) | 3.53 (2.48-4.04) | 1.26-7.12 | 2.56 (2.39-3.70) | 1.58-5.93 | 1.38 |  |
| FA(20:2) | 2.68 (1.79-3.49) | 1.20-9.09 | 2.34 (1.58-2.73) | 1.24-4.20 | 1.14 |  |
| FA(20:3) | 3.68 (2.38-8.14) | 1.59-25.88 | 3.62 (2.75-5.03) | 1.51-8.26 | 1.02 |  |
| **Acylcarnitines** | | | | | | |
| C0 | 20.78 (14.33-40.74) | 10.58-79.66 | 14.81 (13.51-17.95) | 9.42-24.65 | 1.40 |  |
| C2 | 2.81 (1.71-4.36) | 0.74-14.78 | 1.44 (1.08-1.67) | 0.79-2.09 | 1.95* |  |
| C5 | 0.13 (0.10-0.19) | 0.06-0.37 | 0.09 (0.07-0.11) | 0.06-0.15 | 1.39 |  |
| **Cholesterol esters** | | | | | | |
| CE(14:0) | 24.81 (21.4-43.17) | 15.75-68.04 | 15.17 (12.00-17.74) | 7.41-29.68 | 1.64* |  |
| CE(15:0) | 11.69 (9.33-17.31) | 3.86-26.42 | 4.03 (3.32-5.00) | 2.33-9.31 | 2.9* |  |
| CE(16:0) | 171.0 (147.3-234.1) | 104.4-296.4 | 148.1 (130.0-181.9) | 49.35-303.4 | 1.16 |  |
| CE(17:0) | 28.28 (16.04-40.70) | 6.17-58.10 | 8.08 (4.56-11.48) | 1.75-32.63 | 3.5* |  |
| CE(18:0) | 18.05 (13.32-25.21) | 8.79-36.07 | 10.81 (8.47-15.46) | 2.80-35.31 | 1.67* |  |
| CE(18:1) | 447.0 (387.2-610.1) | 198.1-773.2 | 386.4 (247.0-480.9) | 62.39-981.36 | 1.16 |  |
| CE(18:2) | 601.8 (501.1-869.3) | 357.2-1091.5 | 557.08 (417.9-704.3) | 228.9-1120.8 | 1.08 |  |
| CE(18:3) | 54.16 (41.3-70.98) | 27.60-93.80 | 33.48 (24.47-46.34) | 15.16-79.07 | 1.62* |  |
| CE(20:1) | 16.96 (9.03-37.83) | 1.93-73.20 | 19.9 (12.6-31.59) | 7.33-39.43 | -1.17 |  |
| CE(20:3) | 4.44 (3.48-5.07) | 3.00-8.85 | 4.31 (3.31-5.34) | 2.34-11.36 | 1.03 |  |
| CE(20:4) | 46.94 (34.85-82.67) | 20.48-116.7 | 32.92 (22.67-48.16) | 8.67-69.61 | 1.43 |  |
| CE(20:5) | 6.59 (5.28-7.72) | 3.74-11.81 | 5.79 (3.93-7.38) | 2.95-14.48 | 1.14 |  |
| CE(22:6) | 3.65 (2.57-4.74) | 1.90-13.01 | 3.39 (2.75-4.00) | 1.60-7.40 | 1.08 |  |
| **Lysophosphatidylcholines (choline lyso-lecithins)** | | | | | | |
| lysoPC a C16:0 | 160.7 (115.1-195.9) | 28.62-378.0 | 149.5 (118.7-172.5) | 78.83-227.1 | 1.08 |  |
| lysoPC a C16:1 | 2.46 (1.83-2.78) | 0.28-5.74 | 1.77 (1.42-2.00) | 0.93-2.79 | 1.39 |  |
| lysoPC a C17:0 | 10.11 (7.41-12.27) | 1.85-19.58 | 3.13 (2.06-4.34) | 1.26-5.39 | 3.24* |  |
| lysoPC a C18:0 | 163.9 (96.91-200.9) | 62.56-305.5 | 101.5 (72.38-126.0) | 49.72-149.4 | 1.61* |  |
| lysoPC a C18:1 | 62.52 (40.99-68.19) | 14.07-108.1 | 37.05 (27.23-46.99) | 17.39-57.07 | 1.69* |  |
| lysoPC a C18:2 | 19.96 (14.07-23.76) | 1.54-39.73 | 16.00 (12.71-25.67) | 8.29-38.19 | 1.25 |  |
| lysoPC a C20:3 | 0.99 (0.79-1.26) | 0.34-1.64 | 0.69 (0.56-0.94) | 0.45-1.32 | 1.43 |  |
| lysoPC a C20:4 | 3.18 (2.56-4.95) | 0.75-11.66 | 2.56 (2.00-3.71) | 0.89-6.12 | 1.24 |  |
| lysoPC a C28:0 | 0.38 (0.31-0.55) | 0.22-0.94 | 0.33 (0.27-0.36) | 0.23-0.59 | 1.17 |  |
| **Diacyl-phosphatidylcholines (choline lecithins)** | | | | | | |
| PC aa C24:0 | 0.32 (0.22-0.46) | 0.12-0.73 | 0.25 (0.18-0.30) | 0.13-0.41 | 1.27 |  |
| PC aa C28:1 | 1.36 (1.16-1.66) | 0.84-2.44 | 0.99 (0.85-1.24) | 0.80-1.37 | 1.37 |  |
| PC aa C30:0 | 4.98 (4.13-7.36) | 3.19-18.74 | 3.91 (2.81-5.65) | 1.91-6.89 | 1.28 |  |
| PC aa C32:0 | 24.72 (17.5-29.84) | 14.76-73.24 | 23.55 (18.16-26.71) | 14.25-41.08 | 1.05 |  |
| PC aa C32:1 | 9.54 (8.25-12.28) | 5.49-20.58 | 7.50 (5.61-9.83) | 4.03-12.4 | 1.27 |  |
| PC aa C32:2 | 3.85 (2.96-4.73) | 2.19-5.65 | 3.48 (2.00-5.46) | 1.10-7.19 | 1.11 |  |
| PC aa C32:3 | 0.38 (0.34-0.44) | 0.24-0.59 | 0.45 (0.33-0.52) | 0.27-0.67 | -1.19 |  |
| PC aa C34:1 | 143.5 (128.8-168.9) | 67.58-220.2 | 125.32 (100.5-159.2) | 77.71-182.7 | 1.14 |  |
| PC aa C34:2 | 197.5 (167.1-213.4) | 125.9-249.8 | 215.74 (171.8-259.7) | 126.6-343.0 | -1.09 |  |
| PC aa C34:3 | 8.21 (6.38-9.11) | 4.59-12.41 | 7.48 (5.46-9.46) | 3.82-11.32 | 1.10 |  |
| PC aa C34:4 | 0.92 (0.75-1.23) | 0.43-1.82 | 0.58 (0.45-0.72) | 0.34-1.17 | 1.58* |  |
| PC aa C36:0 | 3.26 (2.03-6.28) | 1.18-14.9 | 3.52 (2.25-4.98) | 1.12-9.96 | -1.08 |  |
| PC aa C36:1 | 108.6 (88.2-127.0) | 33.29-194.3 | 55.66 (43.45-76.5) | 33.6-104.57 | 1.95* |  |
| PC aa C36:2 | 219.2 (189.8-255.1) | 120.4-317.1 | 194.89 (161.1-233.1) | 118.2-303.7 | 1.12 |  |
| PC aa C36:3 | 65.09 (54.36-73.92) | 40.68-92.79 | 48.33 (36.36-66.33) | 27.58-86.92 | 1.35 |  |
| PC aa C36:4 | 53.65 (39.11-70.51) | 23.95-93.86 | 39.41 (29.42-55.09) | 23.44-64.27 | 1.36 |  |
| PC aa C36:5 | 4.34 (3.36-5.34) | 2.31-9.00 | 3.74 (2.83-4.86) | 1.98-6.93 | 1.16 |  |
| PC aa C36:6 | 0.23 (0.16-0.29) | 0.07-0.58 | 0.31 (0.24-0.36) | 0.16-0.57 | -1.34 |  |
| PC aa C38:0 | 2.77 (1.15-4.73) | 0.73-20.84 | 3.20 (1.85-4.79) | 1.13-9.60 | -1.15 |  |
| PC aa C38:1 | 1.34 (0.83-2.00) | 0.42-5.63 | 1.41 (1.03-1.99) | 0.65-2.36 | -1.05 |  |
| PC aa C38:3 | 14.54 (12.27-16.61) | 8.28-23.43 | 11.1 (8.62-14.05) | 6.38-19.50 | 1.31 |  |
| PC aa C38:4 | 68.30 (56.2-106.1) | 28.78-131.2 | 46.44 (35.88-67.33) | 25.00-81.83 | 1.47 |  |
| PC aa C38:5 | 22.21 (18.51-28.87) | 11.24-39.2 | 19.06 (13.78-28.93) | 10.27-33.5 | 1.17 |  |
| PC aa C38:6 | 5.75 (4.47-7.79) | 2.34-12.35 | 5.60 (4.24-8.53) | 2.79-11.93 | 1.03 |  |
| PC aa C40:2 | 1.63 (0.92-3.02) | 0.27-5.90 | 2.59 (1.79-3.31) | 1.05-4.30 | -1.59 |  |
| PC aa C40:3 | 1.71 (1.21-2.47) | 0.75-4.10 | 2.15 (1.57-2.40) | 1.17-2.97 | -1.26 |  |
| PC aa C40:4 | 4.24 (3.14-4.93) | 1.88-7.47 | 3.63 (3.31-5.11) | 2.27-7.17 | 1.17 |  |
| PC aa C40:5 | 8.17 (5.52-11.00) | 3.03-19.98 | 6.78 (5.54-10.52) | 3.68-14.35 | 1.20 |  |
| PC aa C40:6 | 5.34 (3.33-7.07) | 1.55-12.22 | 4.29 (3.01-6.35) | 1.78-9.17 | 1.24 |  |
| PC aa C42:0 | 0.31 (0.27-0.59) | 0.10-1.27 | 0.44 (0.33-0.59) | 0.22-0.69 | -1.4 |  |
| PC aa C42:1 | 0.24 (0.19-0.42) | 0.09-0.87 | 0.35 (0.28-0.47) | 0.17-0.55 | -1.46 |  |
| PC aa C42:2 | 0.32 (0.19-0.48) | 0.10-0.84 | 0.51 (0.35-0.55) | 0.27-0.81 | -1.6 |  |
| PC aa C42:4 | 0.38 (0.22-0.70) | 0.12-1.27 | 0.51 (0.47-0.65) | 0.30-1.07 | -1.36 |  |
| PC aa C42:5 | 0.40 (0.29-0.50) | 0.14-0.72 | 0.40 (0.30-0.47) | 0.21-0.65 | 1.00 |  |
| PC aa C42:6 | 0.45 (0.39-0.56) | 0.25-0.81 | 0.41 (0.34-0.52) | 0.28-0.67 | 1.09 |  |
| **Acyl-alkyl-phosphatidylcholines (choline plasmalogens)** | | | | | | |
| PC ae C30:0 | 0.80 (0.73-1.23) | 0.65-2.21 | 0.68 (0.53-0.75) | 0.41-0.99 | 1.18 |  |
| PC ae C30:1 | 0.13 (0.09-0.16) | 0.04-0.26 | 0.14 (0.12-0.18) | 0.10-0.28 | -1.06 |  |
| PC ae C30:2 | 0.10 (0.08-0.13) | 0.04-0.27 | 0.10 (0.08-0.13) | 0.06-0.18 | -1.07 |  |
| PC ae C32:1 | 2.64 (2.45-2.94) | 1.69-5.08 | 2.6 (2.21-2.77) | 1.77-3.86 | 1.01 |  |
| PC ae C32:2 | 0.88 (0.74-1.09) | 0.60-1.29 | 0.84 (0.7-1.00) | 0.55-1.23 | 1.04 |  |
| PC ae C34:0 | 5.18 (3.06-6.42) | 0.77-8.65 | 1.02 (0.86-1.57) | 0.63-1.89 | 5.07* |  |
| PC ae C34:1 | 13.01 (9.60-14.32) | 6.21-17.73 | 7.52 (6.62-8.27) | 4.90-10.26 | 1.73* |  |
| PC ae C34:2 | 7.36 (5.89-8.38) | 5.38-11.79 | 5.72 (5.23-6.38) | 3.72-7.84 | 1.29 |  |
| PC ae C34:3 | 2.10 (1.81-2.79) | 1.13-4.11 | 2.02 (1.83-2.23) | 1.37-3.25 | 1.04 |  |
| PC ae C36:0 | 2.18 (1.45-2.86) | 0.66-3.88 | 0.80 (0.63-1.06) | 0.48-1.55 | 2.72* |  |
| PC ae C36:1 | 20.3 (16.21-25.06) | 6.86-35.35 | 17.54 (13.83-23.3) | 8.52-31.29 | 1.16 |  |
| PC ae C36:2 | 17.93 (13.56-21.51) | 7.16-32.82 | 10.13 (7.6-13) | 5.22-14.05 | 1.77* |  |
| PC ae C36:3 | 4.29 (3.61-4.84) | 2.54-6.21 | 3.08 (2.64-3.28) | 1.86-3.85 | 1.39 |  |
| PC ae C36:4 | 3.77 (2.76-4.96) | 1.94-7.68 | 2.59 (2.18-3.03) | 1.55-4.27 | 1.46 |  |
| PC ae C36:5 | 2.31 (1.56-3.51) | 0.86-5.74 | 1.66 (1.37-2.21) | 0.93-2.70 | 1.39 |  |
| PC ae C38:0 | 0.72 (0.61-1.04) | 0.35-2.62 | 0.64 (0.47-0.76) | 0.36-1.03 | 1.13 |  |
| PC ae C38:1 | 10.97 (6.81-19.06) | 2.43-31.41 | 13.67 (11.02-19.31) | 7.77-23.43 | -1.25 |  |
| PC ae C38:2 | 7.33 (6.4-12.09) | 3.14-20.81 | 8.70 (6.78-9.79) | 3.64-11.50 | -1.19 |  |
| PC ae C38:3 | 7.32 (5.71-8.84) | 3.27-19.84 | 8.68 (7.07-9.83) | 4.37-13.63 | -1.19 |  |
| PC ae C38:4 | 6.8 (4.61-8.35) | 2.28-12.56 | 3.54 (2.42-4.38) | 1.78-5.29 | 1.92* |  |
| PC ae C38:5 | 3.95 (2.65-4.51) | 1.45-7.08 | 2.42 (1.85-3.17) | 1.19-3.98 | 1.63* |  |
| PC ae C38:6 | 1.99 (1.37-2.51) | 0.71-4.77 | 1.38 (1.07-1.82) | 0.70-2.55 | 1.44 |  |
| PC ae C40:1 | 0.73 (0.57-1.03) | 0.36-2.02 | 0.89 (0.65-1.00) | 0.40-1.36 | -1.22 |  |
| PC ae C40:2 | 1.39 (1.14-1.80) | 0.56-3.25 | 1.60 (1.30-1.80) | 0.81-2.34 | -1.15 |  |
| PC ae C40:3 | 6.64 (3.43-9.35) | 2.00-23.38 | 7.58 (6.7-10.21) | 5.65-14.15 | -1.14 |  |
| PC ae C40:4 | 4.45 (3.30-6.23) | 1.96-11.8 | 4.64 (4.18-6.29) | 3.19-9.36 | -1.04 |  |
| PC ae C40:5 | 2.57 (1.91-3.05) | 1.00-5.87 | 2.17 (1.82-2.62) | 1.56-4.70 | 1.18 |  |
| PC ae C40:6 | 1.06 (0.79-1.50) | 0.12-3.51 | 0.68 (0.54-0.79) | 0.38-1.34 | 1.56* |  |
| PC ae C42:1 | 0.78 (0.53-1.30) | 0.29-2.92 | 0.92 (0.82-1.43) | 0.46-1.73 | -1.19 |  |
| PC ae C42:2 | 0.56 (0.42-0.95) | 0.21-2.01 | 0.81 (0.64-1.07) | 0.37-1.31 | -1.43 |  |
| PC ae C42:3 | 0.76 (0.50-1.09) | 0.19-1.93 | 1.06 (0.89-1.17) | 0.75-1.69 | -1.39 |  |
| PC ae C42:4 | 1.36 (0.51-2.12) | 0.25-3.50 | 1.58 (1.42-2.06) | 1.13-3.60 | -1.16 |  |
| PC ae C42:5 | 1.46 (1.09-2.01) | 0.75-3.40 | 1.45 (1.28-1.67) | 1.02-2.97 | 1.01 |  |
| PC ae C44:3 | 0.30 (0.22-0.46) | 0.13-0.91 | 0.37 (0.27-0.43) | 0.22-0.56 | -1.24 |  |
| PC ae C44:5 | 0.44 (0.30-0.52) | 0.20-0.73 | 0.32 (0.24-0.38) | 0.20-0.45 | 1.36 |  |
| PC ae C44:6 | 0.22 (0.19-0.32) | 0.12-0.45 | 0.24 (0.20-0.28) | 0.13-0.33 | -1.06 |  |
| **Sphingomyelins** | | | | | | |
| SM (OH) C14:1 | 2.88 (2.36-3.64) | 1.76-4.97 | 1.88 (1.57-2.09) | 1.28-2.67 | 1.53* |  |
| SM (OH) C16:1 | 3.03 (2.14-3.78) | 1.20-5.53 | 1.25 (1.16-1.65) | 0.81-2.28 | 2.42* |  |
| SM (OH) C22:1 | 2.10 (1.82-2.5) | 1.29-3.33 | 1.93 (1.69-2.18) | 1.24-3.23 | 1.09 |  |
| SM (OH) C22:2 | 1.05 (0.90-1.23) | 0.61-1.63 | 0.86 (0.65-1.03) | 0.49-1.91 | 1.22 |  |
| SM (OH) C24:1 | 0.57 (0.47-0.65) | 0.34-0.89 | 0.47 (0.37-0.58) | 0.25-0.68 | 1.23 |  |
| SM C16:0 | 50.32 (45.81-54.42) | 30.05-77.02 | 44.13 (37.9-51.72) | 30.37-60.37 | 1.14 |  |
| SM C16:1 | 4.88 (4.28-6.00) | 3.20-7.33 | 4.08 (3.42-4.63) | 2.92-5.59 | 1.20 |  |
| SM C18:0 | 8.10 (7.17-9.69) | 3.89-12.29 | 6.09 (5.10-7.18) | 4.30-9.92 | 1.33 |  |
| SM C18:1 | 3.60 (2.98-4.37) | 2.29-5.41 | 2.59 (2.34-3.16) | 1.93-4.53 | 1.39 |  |
| SM C20:2 | 0.13 (0.09-0.16) | 0.07-0.20 | 0.08 (0.06-0.10) | 0.06-0.18 | 1.64* |  |
| SM C24:0 | 4.24 (3.30-4.92) | 2.29-5.95 | 4.37 (3.89-4.73) | 2.65-7.55 | -1.03 |  |
| SM C24:1 | 6.81 (5.55-7.88) | 4.58-11.00 | 6.58 (5.77-7.65) | 4.29-17.52 | 1.03 |  |
| **Ceramides** | | | | | | |
| Cer(d16:1/23:0) | 0.10 (0.07-0.12) | 0.04-0.20 | 0.08 (0.06-0.09) | 0.03-0.11 | 1.24 |  |
| Cer(d16:1/24:0) | 0.08 (0.06-0.10) | 0.05-0.15 | 0.08 (0.05-0.08) | 0.03-0.11 | -1.00 |  |
| Cer(d18:1/16:0) | 0.33 (0.24-0.40) | 0.18-0.56 | 0.21 (0.16-0.23) | 0.14-0.30 | 1.54* |  |
| Cer(d18:1/18:0) | 0.31 (0.19-0.40) | 0.09-0.80 | 0.10 (0.08-0.15) | 0.06-0.22 | 3.06* |  |
| Cer(d18:1/20:0) | 0.07 (0.06-0.11) | 0.04-0.14 | 0.05 (0.04-0.07) | 0.03-0.10 | 1.35 |  |
| Cer(d18:1/22:0) | 0.65 (0.40-0.90) | 0.27-1.33 | 0.37 (0.26-0.48) | 0.19-0.91 | 1.76* |  |
| Cer(d18:1/23:0) | 0.97 (0.61-1.30) | 0.37-2.14 | 0.57 (0.37-0.68) | 0.26-1.33 | 1.69* |  |
| Cer(d18:1/24:0) | 0.72 (0.56-0.89) | 0.37-1.71 | 0.55 (0.34-0.68) | 0.26-1.38 | 1.30 |  |
| Cer(d18:1/24:1) | 0.46 (0.33-0.55) | 0.23-0.98 | 0.29 (0.24-0.40) | 0.16-0.53 | 1.60* |  |
| Cer(d18:1/25:0) | 0.34 (0.27-0.44) | 0.16-0.95 | 0.19 (0.15-0.23) | 0.11-0.38 | 1.80* |  |
| **Glycosphingolipids** | | | | | | |
| HexCer (d18:1/16:0) | 0.50 (0.43-0.63) | 0.34-0.96 | 0.47 (0.41-0.55) | 0.26-0.73 | 1.05 |  |
| HexCer (d18:1/18:1) | 0.05 (0.04-0.05) | 0.03-0.08 | 0.05 (0.04-0.05) | 0.02-0.06 | 1.05 |  |
| HexCer (d18:1/22:0) | 1.15 (0.87-1.39) | 0.67-1.76 | 0.92 (0.75-1.02) | 0.56-1.39 | 1.25 |  |
| HexCer (d18:1/24:0) | 0.45 (0.41-0.59) | 0.30-0.74 | 0.41 (0.33-0.48) | 0.24-0.57 | 1.12 |  |
| HexCer (d18:1/24:1) | 1.35 (1.22-1.52) | 0.84-2.32 | 1.31 (1.14-1.48) | 0.85-1.96 | 1.03 |  |
| Hex2Cer (d18:1/16:0) | 0.69 (0.63-0.74) | 0.46-1.00 | 0.68 (0.58-0.76) | 0.42-0.91 | 1.01 |  |
| **Simple lipids** | | | | | | |
| DG(16:0_18:2) | 1.14 (0.90-1.32) | 0.66-2.18 | 1.06 (0.91-1.43) | 0.40-2.06 | 1.08 |  |
| TG(14:0_32:2) | 1.16 (0.88-2.43) | 0.63-5.32 | 0.81 (0.60-1.21) | 0.27-2.35 | 1.44 |  |
| TG(14:0_34:0) | 5.38 (3.47-8.29) | 1.18-22.57 | 1.85 (1.49-2.45) | 0.68-6.14 | 2.91* |  |
| TG(14:0_34:1) | 18.45 (10.00-31.6) | 5.96-76.82 | 9.68 (6.82-13.28) | 2.15-28.04 | 1.91* |  |
| TG(14:0_34:2) | 3.89 (2.55-7.08) | 1.74-18.65 | 2.89 (2.24-4.11) | 0.69-9.35 | 1.35 |  |
| TG(14:0_36:1) | 5.39 (3.66-8.53) | 1.14-19.66 | 1.84 (1.33-2.35) | 0.60-5.19 | 2.93* |  |
| TG(14:0_36:2) | 8.49 (5.17-13.37) | 2.22-32.54 | 3.88 (3.27-6.10) | 0.85-14.6 | 2.19* |  |
| TG(14:0_36:3) | 2.14 (1.40-3.35) | 1.09-9.32 | 1.70 (1.41-2.85) | 0.32-6.53 | 1.26 |  |
| TG(16:0_28:1) | 9.54 (5.64-19.82) | 1.81-35.78 | 1.77 (1.28-2.20) | 0.53-3.95 | 5.38* |  |
| TG(16:0_28:2) | 1.29 (0.81-2.79) | 0.44-5.48 | 0.44 (0.37-0.53) | 0.11-1.27 | 2.94* |  |
| TG(16:0_30:2) | 1.47 (1.06-4.55) | 0.65-17.32 | 3.60 (2.30-4.43) | 0.60-9.18 | -2.45 |  |
| TG(16:0_32:0) | 26.74 (17.29-53.55) | 10.13-177.3 | 23.78 (14.45-29.83) | 4.76-80.09 | 1.12 |  |
| TG(16:0_32:1) | 23.79 (12.81-36.6) | 8.37-113.27 | 13.28 (8.21-16.34) | 2.97-46.93 | 1.79* |  |
| TG(16:0_32:2) | 3.82 (2.53-6.51) | 1.62-18.93 | 3.39 (2.41-4.52) | 0.68-10.70 | 1.13 |  |
| TG(16:0_32:3) | 0.52 (0.36-0.66) | 0.26-1.77 | 0.45 (0.29-0.59) | 0.16-1.46 | 1.16 |  |
| TG(16:0_33:1) | 5.65 (3.33-8.35) | 0.89-26.52 | 1.12 (0.65-2.05) | 0.36-6.82 | 5.04* |  |
| TG(16:0_33:2) | 1.16 (0.86-1.49) | 0.46-4.75 | 0.57 (0.42-1.16) | 0.25-1.77 | 2.02* |  |
| TG(16:0_34:0) | 14.08 (7.97-17.52) | 6.40-56.67 | 8.60 (6.02-14.79) | 3.85-28.47 | 1.64* |  |
| TG(16:0_34:1) | 52.75 (30.65-95.80) | 15.25-243.21 | 42.37 (30.53-58.53) | 9.12-164.88 | 1.24 |  |
| TG(16:0_34:2) | 14.42 (8.34-25.35) | 5.36-70.18 | 15.00 (10.56-23.47) | 3.69-62.68 | -1.04 |  |
| TG(16:0_34:3) | 1.86 (1.33-3.09) | 0.71-8.51 | 1.73 (1.00-2.64) | 0.51-8.41 | 1.08 |  |
| TG(16:0_35:1) | 6.06 (3.76-8.40) | 0.61-19.47 | 0.97 (0.54-1.35) | 0.27-5.68 | 6.28* |  |
| TG(16:0_35:2) | 3.39 (2.14-4.42) | 0.59-10.61 | 0.98 (0.58-1.33) | 0.22-4.90 | 3.47* |  |
| TG(16:0_35:3) | 0.69 (0.51-0.87) | 0.16-2.15 | 0.40 (0.28-0.66) | 0.15-1.23 | 1.74* |  |
| TG(16:0_36:2) | 30.32 (20.13-49.17) | 7.25-94.31 | 23.49 (16.61-36.68) | 5.73-90.48 | 1.29 |  |
| TG(16:0_36:3) | 10.47 (6.46-17.47) | 4-34.12.00 | 11.85 (9.15-19.23) | 3.01-43.56 | -1.13 |  |
| TG(16:0_36:4) | 2.16 (1.49-3.29) | 0.96-6.80 | 2.54 (1.85-4.12) | 0.82-8.74 | -1.18 |  |
| TG(16:0_38:1) | 0.47 (0.43-0.68) | 0.26-1.62 | 0.53 (0.38-0.62) | 0.19-1.11 | -1.13 |  |
| TG(16:0_38:2) | 0.98 (0.61-1.49) | 0.41-4.79 | 0.77 (0.54-1.00) | 0.28-3.74 | 1.27 |  |
| TG(16:0_38:3) | 0.81 (0.56-1.27) | 0.39-4.86 | 0.66 (0.47-1.00) | 0.28-2.03 | 1.21 |  |
| TG(16:0_38:4) | 0.97 (0.74-1.52) | 0.42-4.97 | 0.91 (0.51-1.26) | 0.42-2.75 | 1.07 |  |
| TG(16:0_38:5) | 0.83 (0.57-1.17) | 0.40-3.35 | 0.83 (0.50-1.59) | 0.19-2.57 | -1.00 |  |
| TG(16:1_28:0) | 1.61 (1.04-2.93) | 0.75-6.27 | 0.98 (0.73-1.13) | 0.43-2.25 | 1.64* |  |
| TG(16:1_30:1) | 0.92 (0.64-1.42) | 0.38-3.24 | 0.74 (0.58-1.01) | 0.34-1.60 | 1.24 |  |
| TG(16:1_32:0) | 3.12 (2.05-5.94) | 1.48-22.27 | 1.94 (0.97-2.77) | 0.46-12.25 | 1.61* |  |
| TG(16:1_32:1) | 2.45 (1.49-3.93) | 0.73-11.69 | 1.02 (0.69-1.78) | 0.40-5.56 | 2.41* |  |
| TG(16:1_34:0) | 1.73 (1.19-2.56) | 0.57-7.11 | 0.71 (0.36-1.11) | 0.24-4.69 | 2.43* |  |
| TG(16:1_34:1) | 6.82 (3.78-9.11) | 2.57-31.38 | 3.69 (2.03-5.77) | 0.87-23.76 | 1.85* |  |
| TG(16:1_34:2) | 1.71 (1.14-2.34) | 0.79-7.72 | 1.45 (0.85-2.26) | 0.47-8.60 | 1.18 |  |
| TG(16:1_36:1) | 1.20 (0.83-2.21) | 0.37-5.18 | 0.67 (0.42-1.36) | 0.20-3.31 | 1.80* |  |
| TG(16:1_36:2) | 2.66 (1.70-4.01) | 1.03-11.33 | 1.83 (1.10-2.69) | 0.65-9.90 | 1.46 |  |
| TG(16:1_36:3) | 0.94 (0.66-1.39) | 0.43-3.57 | 0.90 (0.52-1.45) | 0.34-4.45 | 1.05 |  |
| TG(17:0_34:1) | 5.72 (3.66-7.85) | 0.77-18.22 | 0.76 (0.53-1.22) | 0.23-4.34 | 7.52* |  |
| TG(17:0_34:2) | 1.39 (0.89-1.95) | 0.36-4.65 | 0.52 (0.41-0.81) | 0.30-1.60 | 2.68* |  |
| TG(17:1_34:1) | 2.52 (1.25-3.29) | 0.41-8.89 | 0.8 (0.37-2.05) | 0.23-3.40 | 3.13* |  |
| TG(18:0_30:0) | 5.68 (3.99-10.11) | 1.22-25.69 | 1.92 (1.40-5.13) | 1.00-5.92 | 2.97* |  |
| TG(18:0_30:1) | 2.93 (1.95-4.82) | 0.46-9.09 | 1.70 (1.15-2.26) | 0.30-4.22 | 1.72* |  |
| TG(18:0_32:1) | 6.60 (3.82-11.63) | 1.03-25.61 | 1.52 (0.93-2.19) | 0.55-7.00 | 4.35* |  |
| TG(18:0_32:2) | 1.18 (0.83-1.79) | 0.42-4.32 | 0.70 (0.48-1.24) | 0.29-2.14 | 1.70* |  |
| TG(18:0_34:2) | 3.25 (2.35-5.05) | 1.00-11.44 | 1.95 (1.27-3.21) | 0.63-10.27 | 1.66* |  |
| TG(18:0_34:3) | 0.70 (0.43-0.81) | 0.33-2.28 | 0.50 (0.30-0.71) | 0.11-2.03 | 1.41 |  |
| TG(18:0_36:3) | 3.09 (2.34-4.52) | 0.72-10.08 | 2.38 (1.50-3.78) | 0.74-10.24 | 1.30 |  |
| TG(18:0_36:4) | 0.90 (0.62-1.08) | 0.39-2.01 | 0.68 (0.53-1.15) | 0.27-2.52 | 1.31 |  |
| TG(18:1_26:0) | 18.47 (9.57-31.70) | 2.66-60.48 | 4.63 (3.39-6.36) | 0.63-11.00 | 3.99* |  |
| TG(18:1_28:1) | 7.16 (4.65-13.38) | 0.93-29.76 | 1.47 (1.01-1.99) | 0.75-6.27 | 4.88* |  |
| TG(18:1_30:0) | 26.27 (13.93-40.81) | 6.75-87.22 | 12.38 (8.95-17.57) | 2.39-35.23 | 2.12* |  |
| TG(18:1_30:1) | 10.52 (5.65-17.59) | 2.03-48.50 | 10.93 (6.44-13.81) | 1.26-28.26 | -1.04 |  |
| TG(18:1_30:2) | 1.27 (0.70-2.46) | 0.50-9.20 | 2.04 (1.39-2.53) | 0.28-5.21 | -1.61 |  |
| TG(18:1_31:0) | 6.22 (4.11-8.74) | 0.90-25.94 | 1.22 (0.73-2.28) | 0.55-5.17 | 5.09* |  |
| TG(18:1_32:0) | 40.45 (23.43-68.79) | 9.84-151.6 | 28.06 (18.63-35.52) | 6.47-107.47 | 1.44 |  |
| TG(18:1_32:1) | 27.16 (14.82-35.99) | 5.91-93.55 | 11.84 (7.37-15.18) | 3.23-46.49 | 2.29* |  |
| TG(18:1_32:2) | 3.80 (2.20-5.68) | 1.42-14.01 | 2.60 (1.92-3.57) | 0.62-8.41 | 1.46 |  |
| TG(18:1_33:0) | 6.64 (4.33-9.27) | 0.55-22.67 | 0.85 (0.51-1.43) | 0.28-4.85 | 7.84* |  |
| TG(18:1_33:1) | 6.49 (3.51-8.31) | 0.81-26.53 | 1.27 (0.61-1.69) | 0.35-6.83 | 5.12* |  |
| TG(18:1_33:2) | 1.00 (0.71-1.32) | 0.34-4.34 | 0.50 (0.37-0.98) | 0.22-1.40 | 1.99* |  |
| TG(18:1_34:1) | 67.46 (43.14-103.6) | 13.07-218.6 | 47.49 (33.87-72.82) | 11.18-192.47 | 1.42 |  |
| TG(18:1_34:2) | 15.85 (9.39-22.00) | 5.46-51.50 | 14.59 (10.58-22.9) | 3.81-58.09 | 1.09 |  |
| TG(18:1_34:3) | 1.58 (1.07-2.64) | 0.75-7.06 | 1.60 (0.81-2.18) | 0.41-7.43 | -1.01 |  |
| TG(18:1_35:2) | 3.16 (1.72-4.04) | 0.56-11.24 | 1.01 (0.58-2.46) | 0.23-4.23 | 3.13* |  |
| TG(18:1_36:3) | 8.74 (5.09-11.26) | 3.83-26.44 | 9.07 (6.85-10.48) | 3.65-30.43 | -1.04 |  |
| TG(18:1_36:4) | 1.68 (1.14-2.24) | 0.76-4.70 | 1.73 (1.10-2.54) | 0.83-5.96 | -1.03 |  |
| TG(18:1_36:5) | 0.38 (0.31-0.45) | 0.18-0.77 | 0.36 (0.26-0.41) | 0.14-0.87 | 1.06 |  |
| TG(18:1_38:5) | 0.83 (0.56-1.28) | 0.32-1.99 | 0.66 (0.50-0.90) | 0.32-2.15 | 1.27 |  |
| TG(18:2_28:0) | 2.86 (1.72-6.96) | 0.98-22.29 | 4.54 (2.71-5.67) | 0.45-11.82 | -1.59 |  |
| TG(18:2_30:0) | 4.15 (2.57-6.85) | 1.72-18.44 | 3.73 (2.33-5.42) | 0.78-10.83 | 1.11 |  |
| TG(18:2_30:1) | 1.39 (0.84-3.13) | 0.51-9.47 | 2.02 (1.48-2.95) | 0.26-5.73 | -1.46 |  |
| TG(18:2_31:0) | 1.29 (0.96-2.01) | 0.45-5.23 | 0.68 (0.51-1.43) | 0.35-1.54 | 1.89* |  |
| TG(18:2_32:0) | 7.1 (3.77-12.09) | 2.53-29.85 | 7.74 (5.71-12.39) | 1.99-29.47 | -1.09 |  |
| TG(18:2_32:1) | 3.35 (2.44-7.17) | 1.74-15.03 | 2.83 (1.85-4.16) | 0.71-9.78 | 1.19 |  |
| TG(18:2_32:2) | 0.70 (0.50-1.26) | 0.37-2.40 | 0.74 (0.54-0.98) | 0.23-1.88 | -1.06 |  |
| TG(18:2_33:1) | 1.01 (0.68-1.32) | 0.26-4.08 | 0.46 (0.36-0.99) | 0.17-1.40 | 2.21* |  |
| TG(18:2_34:0) | 3.54 (2.50-5.81) | 1.18-12.06 | 2.83 (1.82-4.67) | 0.93-11.68 | 1.25 |  |
| TG(18:2_34:1) | 12.16 (7.65-20.38) | 4.69-38.66 | 12.82 (9.97-20.36) | 3.48-47.88 | -1.05 |  |
| TG(18:2_34:2) | 2.85 (1.86-5.02) | 1.27-9.68 | 3.83 (2.79-6.57) | 1.06-14.53 | -1.34 |  |
| TG(18:2_35:1) | 1.29 (0.73-1.64) | 0.30-3.88 | 0.51 (0.32-1.06) | 0.17-1.47 | 2.52* |  |
| TG(18:2_36:0) | 0.71 (0.50-0.94) | 0.17-2.06 | 0.68 (0.39-0.76) | 0.34-1.42 | 1.05 |  |
| TG(18:2_36:1) | 3.26 (2.27-4.90) | 1.01-10.52 | 2.39 (1.51-4.08) | 0.73-9.61 | 1.36 |  |
| TG(18:2_36:2) | 4.47 (2.70-6.18) | 1.81-13.78 | 4.67 (3.20-6.36) | 1.99-17.47 | -1.04 |  |
| TG(18:3_34:1) | 1.09 (0.78-1.64) | 0.51-3.87 | 1.16 (0.64-1.43) | 0.30-3.93 | -1.06 |  |
| TG(18:3_36:2) | 0.45 (0.36-0.65) | 0.19-1.26 | 0.55 (0.39-0.60) | 0.19-1.68 | -1.21 |  |
| TG(20:1_34:1) | 0.94 (0.58-1.53) | 0.40-4.45 | 0.82 (0.62-1.17) | 0.30-3.51 | 1.15 |  |
| TG(20:2_34:1) | 0.63 (0.43-0.99) | 0.17-3.54 | 0.49 (0.25-0.69) | 0.11-1.11 | 1.28 |  |
| TG(20:3_32:0) | 1.04 (0.78-1.63) | 0.45-7.42 | 0.73 (0.47-1.04) | 0.18-2.10 | 1.42 |  |
| TG(20:3_32:1) | 0.64 (0.45-0.96) | 0.22-3.42 | 0.49 (0.35-0.73) | 0.11-1.12 | 1.31 |  |
| TG(20:3_34:1) | 2.20 (1.40-3.44) | 0.74-10.43 | 1.34 (0.75-2.36) | 0.29-3.82 | 1.64* |  |
| TG(20:3_34:2) | 0.68 (0.44-1.19) | 0.18-3.42 | 0.57 (0.40-0.85) | 0.15-1.18 | 1.19 |  |
| TG(20:3_36:3) | 0.53 (0.44-0.69) | 0.22-2.59 | 0.46 (0.29-0.63) | 0.26-1.00 | 1.16 |  |
| TG(20:4_30:0) | 0.64 (0.47-0.78) | 0.23-3.02 | 0.44 (0.29-0.61) | 0.20-1.13 | 1.46 |  |
| TG(20:4_32:0) | 1.07 (0.82-1.46) | 0.39-5.10 | 1.09 (0.60-1.69) | 0.29-4.05 | -1.02 |  |
| TG(20:4_32:1) | 0.67 (0.36-0.95) | 0.23-1.93 | 0.49 (0.29-0.69) | 0.19-1.15 | 1.36 |  |
| TG(20:4_34:0) | 0.71 (0.52-0.97) | 0.26-2.52 | 0.64 (0.48-0.84) | 0.17-1.84 | 1.11 |  |
| TG(20:4_34:1) | 1.78 (1.17-3.01) | 0.62-6.07 | 1.52 (0.85-2.80) | 0.46-5.59 | 1.18 |  |
| TG(20:4_34:2) | 0.71 (0.47-1.06) | 0.34-1.94 | 0.85 (0.47-1.19) | 0.20-2.39 | -1.19 |  |
| TG(20:4_36:2) | 1.00 (0.66-1.59) | 0.31-2.41 | 0.77 (0.50-1.13) | 0.30-3.08 | 1.31 |  |
| TG(20:4_36:3) | 0.58 (0.33-0.78) | 0.20-1.12 | 0.54 (0.39-0.72) | 0.19-1.49 | 1.07 |  |
| TG(22:5_32:0) | 0.53 (0.40-0.68) | 0.28-2.59 | 0.43 (0.34-0.66) | 0.15-1.71 | 1.22 |  |
| TG(22:5_34:1) | 0.61 (0.40-0.79) | 0.34-1.85 | 0.61 (0.39-0.74) | 0.20-1.78 | -1.01 |  |

^a^ positive when median from the group left with mothers > median from the group weaned immediately after birth; otherwise negative

* metabolites included in further analysis on the basis of FC_median_ criterion of >1.5
