# Supplementary figures and images for: Profile of serum lipid metabolites of one-week-old goat kids depending on the type of rearing
Source: BMC Vet Res. 2020 Sep 21;16:346. doi: 10.1186/s12917-020-02575-1 (PMC7507259; doi:10.1186/s12917-020-02575-1)

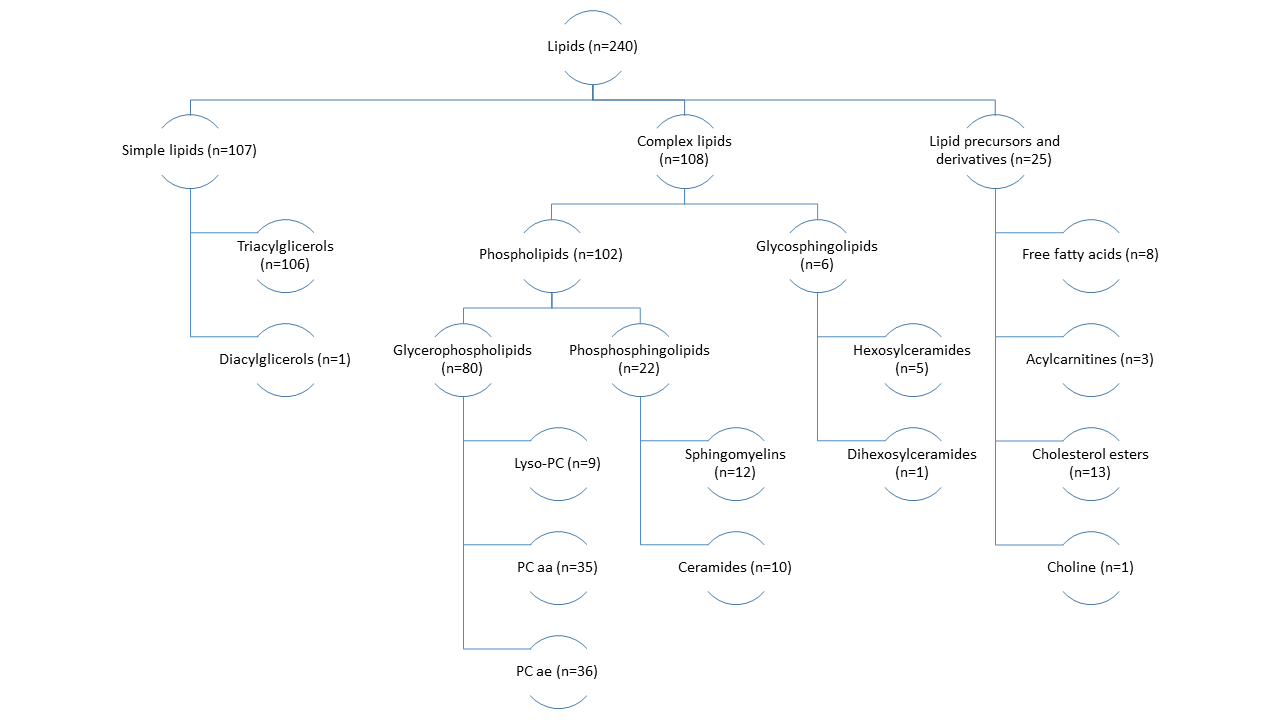

Supplement: Supplementary file 5 — Additional file 5: Figure S5. Classification of lipid metabolites analyzed in this study. Graph showing the type of lipid metabolite classification used in this study. [file 12917_2020_2575_MOESM5_ESM.tif]
